# Advancing PROTAC therapeutics through chemistry-guided design of smart delivery systems

**DOI:** 10.1038/s41401-026-01853-2

**Published:** 2026-06-25

**Authors:** Ting-ting Yao, Zheng Zhou, Guang-ji Wang, Zhe-ying Zhu, Xi-nuo Li

**Affiliations:** 1 State Key Laboratory of Natural Medicines, China Pharmaceutical University, Nanjing, 211198 China; 2 Department of Computer Science, RWTH Aachen University, Aachen, 52074 Germany; 3 School of Pharmacy, The University of Nottingham, Nottingham, NG7 2RD UK

**Keywords:** proteolysis targeting chimeras (PROTACs), delivery systems, chemistry-guided design, prodrugs, nanoparticles

## Abstract

Proteolysis-targeting chimeras (PROTACs) have redefined the paradigm of targeted protein degradation by hijacking the endogenous ubiquitin-proteasome system. However, their clinical translation remains hampered by “beyond-rule-of-five” (bRo5) physicochemical liabilities, including high polarity, limited membrane permeability, and unpredictable pharmacokinetic behavior, which collectively lead to suboptimal bioavailability and risks of non-specific systemic distribution. This review focuses on the chemistry-driven evolution of delivery strategies to transform these molecular liabilities into pharmacological advantages. By integrating medicinal chemistry with delivery science, we propose a conceptual framework to optimize target selectivity, enhance cellular uptake, and broaden the therapeutic window, providing systematic guidance for overcoming the bottlenecks of PROTAC in vivo applications. In terms of molecular design, innovations in prodrug and linker engineering, including photo-responsive, enzyme-activated, folate-targeted, and reactive oxygen species (ROS)-triggered systems, achieve precise spatiotemporal regulation and controlled release of PROTAC activity. The field of delivery vehicles encompasses lipid-based, polymeric, and inorganic nanoscaffolds, as well as antibody-PROTAC conjugates (DACs) and aptamer-PROTAC hybrids (APCs), providing multidimensional platforms for crossing biological barriers and achieving antigen-dependent targeting. Furthermore, the emergence of modular self-assembly, membrane-targeting strategies, and “split-and-hybrid” paradigms signifies a shift toward programmable medicinal chemistry that integrates physicochemical tuning with pharmacokinetic optimization. Future integration of chemically reconfigurable carriers with unified PK-PD evaluation systems will further accelerate the clinical translation of next-generation intelligent PROTAC therapeutics.

**Figure:**
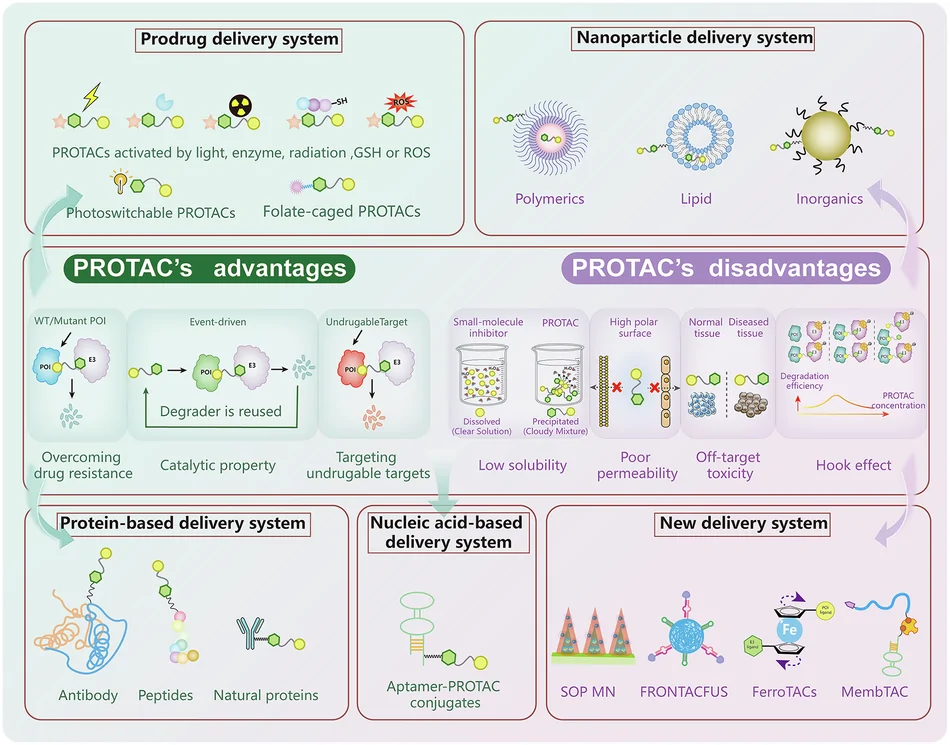
Challenges and innovative strategies in PROTAC delivery system design. PROTAC is regarded as a novel therapeutic strategy with unlimited potential. However, the poor water solubility and low cell permeability of PROTAC result in poor pharmacokinetic properties, thereby limiting the clinical application of PROTAC. The development of PROTAC delivery systems can accelerate the pace of their transformation from experimental to clinical use. These delivery systems can optimize the physicochemical properties of PROTAC, achieve targeted delivery, promote intracellular accumulation and enhance its degradation efficacy.

Challenges and innovative strategies in PROTAC delivery system design. PROTAC is regarded as a novel therapeutic strategy with unlimited potential. However, the poor water solubility and low cell permeability of PROTAC result in poor pharmacokinetic properties, thereby limiting the clinical application of PROTAC. The development of PROTAC delivery systems can accelerate the pace of their transformation from experimental to clinical use. These delivery systems can optimize the physicochemical properties of PROTAC, achieve targeted delivery, promote intracellular accumulation and enhance its degradation efficacy.

## Introduction

One of the core challenges of drug development is that it is difficult to effectively target a large number of disease-related “undruggable” proteins with conventional small molecules or biological agents. These proteins usually lack a clearly defined binding bag or enzyme domain, preventing the participation of stable inhibitors, thus limiting the development of treatment [1–3]. It is estimated that nearly 80% of disease-related proteins in the human proteome belong to this “undruggable” category, which highlights the urgent need for innovative strategies to overcome this bottleneck [4, 5].

**Fig. 1:**
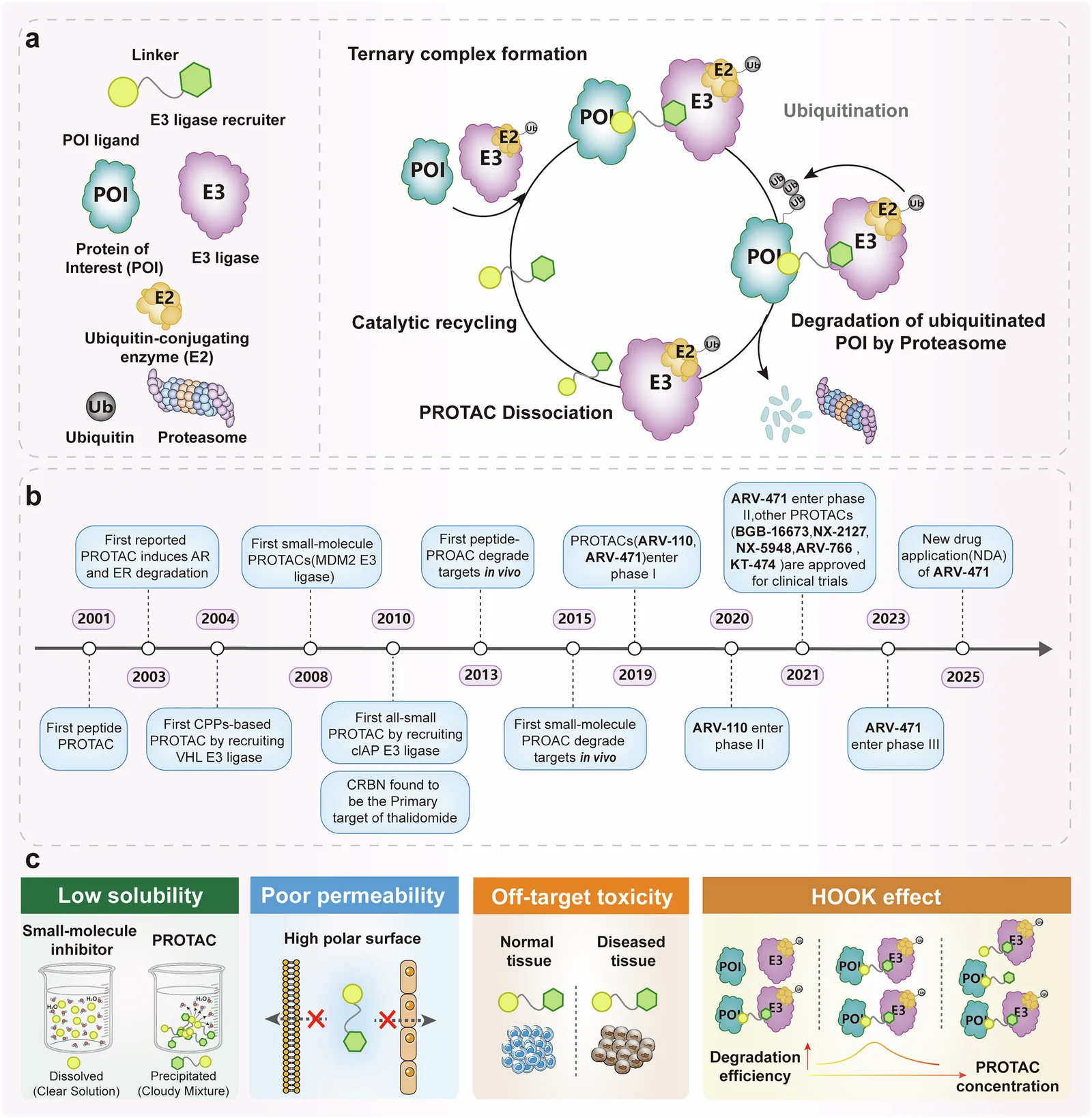
The mechanism, development history and limitations of PROTAC technology. This figure summarizes the basic working mechanism, key developmental milestones and major limitations of PROTACs. **a** PROTAC is composed of interested protein (POI) ligands, linkers and E3 binding enzyme recruiters, which promote the formation of a ternary complex between POI, E3 binding enzyme and E2 (ubiquitin conjugate enzyme) that carries ubiquitin (Ub). This leads to POI ubiquitination, which is then degraded by the proteasome, and the E3 binding enzyme is catalysed and recovered after PROTAC dissolving. **b** PROTAC technology was proposed in 2001 and has gone through key stages such as early peptide verification (2001), breakthrough in small molecule development (2008) and E3 ligand expansion (2010–2015). It entered the clinical transition period in 2019. By 2025, more than 40 drugs are being developed clinically, covering a wide range of diseases and steadily promoting clinical application. **c** PROTAC’s inherent high molecular weight, large polarity surface area (PSA), high logP value and off-target toxicity seriously limit its efficient delivery. The hook effect hinders the degradation efficiency of the target protein.

In order to meet this challenge, Proteolysis-Targeting Chimeras (PROTACs) have become a transformative method for targeted protein degradation (TPD) [6–9]. PROTAC is a dual-functional molecule composed of the ligand of the protein of interest and the ligand of the E3 ubiquitin-binding enzyme, which is connected by a suitable linker. Notably, the PROTACs that have advanced into clinical studies are, in most cases, still based on this small-molecule design paradigm, rather than on macromolecular or biomacromolecular carrier systems. They use the endogenous Ubiquitin-Proteasome System (UPS) to induce selective ubiquitination of target proteins and subsequent proteasome degradation (Fig. 1a) [10–18]. Compared with traditional occupancy drive inhibitors, PROTAC has several key advantages. First of all, they rely on catalytic protein removal instead of inhibiting activity to degrade “undruggable” proteins. Secondly, they act in an event-driven way, that is, a single molecule can catalyze multiple rounds of degradation, achieve substoichiometric efficiency and reduce dose requirements [19–22]. Thirdly, PROTAC can overcome the accessive drug resistance caused by mutation or overexpression by completely removing the pathogenic proprotein instead of blocking it for a short time [3, 11, 23–27]. Overall, these attributes make PROTAC a powerful platform to address the medium and long-term limitations of traditional drug discovery.

Although remarkable progress has been made in drug discovery based on PROTAC, clinical translation is still hindered by inherent structural constraints (Fig. 1a). Traditional PROTAC has a high molecular weight (usually more than 700 Da) and obvious polarity, which severely limits the permeability of passive membranes [28–30]. In addition, their poor water solubility leads to instability in biological culture medium, and low serum stability and limited cell uptake further damage pharmacokinetic properties and bioavailability, eventually leading to non-specific distribution to normal tissues and treatment failure [31–33]. It is reported that due to these delivery obstacles, more than 70% of PROTAC candidates failed to exceed the preclinical evaluation [34, 35]. Therefore, the development of efficient and biocompatible drug delivery systems (DDS) is crucial to realizing the full clinical potential of PROTAC therapy.

It is encouraging that the rapid development of drug delivery technology has opened up new opportunities to overcome these inherent limitations. PROTAC is encapsulated in the conveyor carrier, which can be transported accurately and effectively to the diseased tissue. Such systems reduce non-specific interactions with biological components, thus limiting out-of-target accumulation and minimizing adverse effects. In addition, they allow the space-time control to release PROTAC at the intended site of action, which significantly enhances the therapeutic effect [36–40]. In recent years, various conveying platforms, including liposomes, bionic carriers and antibody-drug conjugates (ADCs), have been explored as carriers for PROTAC. These platforms improve water solubility and cell permeability, optimize the pharmacokinetic characteristics of PROTAC, and promote selective localization in tumor tissue through passive or active targeting strategies, thus significantly enhancing treatment selectivity and treatment window [41–44].

Against this background, this review comprehensively summarises the latest progress of PROTAC-oriented drug delivery strategies and their basic chemical design principles. Unlike existing reviews that primarily discuss PROTAC biology, degrader chemistry, or delivery systems in a more general manner, this review focuses specifically on the chemistry-guided design logic of smart delivery systems for PROTACs and on how the unique structural and physicochemical features of PROTACs impose distinct delivery requirements. By clarifying how the delivery system can work together to improve cell permeability, pharmacokinetic properties and target selectivity, it establishes a conceptual and methodological framework for the design of next-generation degradants with improved drug-like properties. In addition to summarising the current progress, this review also emphasises the emerging integration of medicinal chemistry and delivery science as the basis for the development of intelligent, safe and clinically convertible PROTAC therapies, so as to promote the evolution of precise protein degradation to a mature drug discovery paradigm.

## The development history and clinical translation challenges of PROTAC

### The development history of PROTAC

As an emerging TPD strategy, PROTAC technology harnesses the UPS to selectively degrade disease-associated proteins, offering a novel therapeutic modality for targets traditionally considered “undruggable” by conventional small molecules. The evolution of this technology from concept to clinical application can be delineated into three major phases (Fig. 1b). The conceptual foundation was laid in 2001 by Crews and colleagues, who devised the first peptide-based PROTAC-1 to validate target protein degradation via in vitro E3 recruitment [7]. Subsequent applications targeting estrogen and androgen receptors expanded their scope [45], yet early peptide-dependent PROTACs suffered from high molecular weight and limited membrane permeability. A pivotal shift occurred around 2004 with the discovery of small-molecule E3 ligase ligands [46], culminating in 2008 when Crews’ team developed the first small-molecule PROTAC targeting AR using an MDM2 inhibitor, formally coining the term “PROTAC” and establishing its drug-like potential [47].

The subsequent half-decade witnessed accelerated technological maturation. Notably, the identification of thalidomide and its analogs as CRBN ligands vastly expanded the E3 ligase toolbox [48], spurring a wave of VHL- and CRBN-based PROTACs such as ARV-825 and DBET1 that achieved potent degradation of BRD4 and AR in cellular models [49, 50]. Concurrently, in vivo efficacy emerged: peptide-based PhosphoPROTACs demonstrated 40% tumor inhibition in 2013 [51], and an ERRα-targeting PROTAC reduced tumor target levels by 39% in 2015 [52], collectively signaling the field’s transition toward a self-sustaining research and development ecosystem.

Since 2019, PROTAC technology has entered a phase of clinical translation. Arvinas initiated this era by advancing ARV-110 (AR-targeted) and ARV-471 (ER-targeted) into clinical trials, marking the first in-human evaluation of PROTACs. Early data released in 2020 confirmed their safety, on-target activity, and degradation efficacy [53–56], catalyzing global clinical development. By late 2025, over 40 PROTAC candidates had entered clinical evaluation across oncology, autoimmune disorders, and neurodegeneration (Table 1), targeting proteins including AR, ER, BTK, and IRAK4 [57, 58]. While most programs remain in Phase 1/2, reflecting the field’s relative infancy, several frontrunners have advanced to Phase 2 and beyond, underscoring steady clinical progress [59]. Critically, between 2023 and 2024, multiple leading assets entered pivotal trials.

**Table 1:** PROTACs entering the clinical stage.

PROTAC	Investigational Institution	Target	Indication	Clinical phase
ARV-471	Arvinas / Pfizer	ER	ER-positive/HER2-negative Breast Cancer	NDA
CC-94676	Bristol Myers Squibb	AR	Metastatic castration-resistant prostate cancer	Phase 3
BGB-16673	BeiGene	BTK	B-cell Malignancies/Hematological Tumors	Phase 3
HRS-5041	Hengrui	AR	Metastatic castration-resistant prostate cancer	Phase 2
ARV-766	Arvinas / Novartis	AR	Metastatic castration-resistant prostate cancer	Phase 2
ARV-110	Arvinas	AR	Metastatic castration-resistant prostate cancer	Phase 2
HP-568	Hycreat	ER	ER-positive/HER2-negative Breast Cancer	Phase 2
NX-5948	Nurix	BTK	B-cell Malignancies	Phase 2
KT-621	Kymera	STAT6	Atopic Dermatitis	Phase 2
BGB-45035	BeiGene	IRAK4	Rheumatoid Arthritis	Phase 2
KT-474	Thermo Fisher Scientific / Kymera	IRAK4	Atopic Dermatitis	Phase 2
MT-4561	Tanabe Pharma America	BRD4	NUT Carcinoma	Phase 2
CFT-8634	C4	BRD9	Solid Tumors	Phase 2
PRT-3789	Prelude	SMARCA2	Non-Small Cell Lung Cancer	Phase 2
GT-20029	Kintor	AR	Androgenetic Alopecia	Phase 1/2
TQB3201	Chia Tai Tianqing	AR	Metastatic castration-resistant prostate cancer	Phase 1/2
HP518	Hycreat	AR	Metastatic castration-resistant prostate cancer	Phase 1/2
QLH-12016	Qilu	AR	Metastatic castration-resistant prostate cancer	Phase 1/2
LT-002	Leadingtac	IRAK4	Atopic Dermatitis	Phase 1/2
ARV-806	Arvinas	KRAS G12D	Pancreatic Cancer, Colorectal Cancer and other Solid Tumors	Phase 1/2
DT2216	Dialectic	BCL-XL	T-cell Lymphoma	Phase 1/2
CG 001419	Cullgen	NTRK	Advanced Solid Tumors	Phase 1/2
HSK-38008	Haisco	AR	Metastatic castration-resistant prostate cancer	Phase 1
AC-0176	Accutar	AR	Metastatic castration-resistant prostate cancer	Phase 1
SHR-3591	Hengrui	AR	Metastatic castration-resistant prostate cancer	Phase 1
AH-001	Anhong	AR	Androgenetic Alopecia	Phase 1
TQB-3019	Chia Tai Tianqing	BTK	B-cell Malignancies/Hematological Tumors	Phase 1
AC-0676	Accutar	BTK	B-cell Malignancies/Hematological Tumors	Phase 1
UBX-303-1	Ubix	BTK	B-cell Malignancies/Hematological Tumors	Phase 1
GS-6791	Nurix / Gilead	IRAK4	Atopic Dermatitis	Phase 1
ASP-3082	Astellas	KRAS G12D	Colorectal Cancer	Phase 1
ASP-4396	Astellas	KRAS G12D	Colorectal Cancer	Phase 1
PT-0253	PAQ	KRAS G12D	Advanced Solid Tumors	Phase 1
XNW-34017	Evopoint BioSciences	Aurora A/c-Myc	Malignant Solid Tumors	Phase 1
CFT-1946	C4	BRAF-V600E/BTK	BRAF-mutant Solid Tumors	Phase 1
ARV-393	Arvinas	BCL6	Advanced Non-Hodgkin Lymphoma	Phase 1
CFT-8919	C4	EGFR L858R	EGFR-mutant Non-Small Cell Lung Cancer	Phase 1
HSK-40118	Haisco	EGFR	EGFR-positive Non-Small Cell Lung Cancer	Phase 1
AXT-1003	Axtertx	EZH2	Advanced Malignant Solid Tumors	Phase 1
ARV-102	Arvinas	LRRK2	Neurodegenerative Diseases	Phase 1
AC-0676	Accutar	BTK C481S	B-cell Malignancies/Hematological Tumors	Phase 1
TQB3142	Chia Tai Tianqing	BCL-XL	B-cell Malignancies/Hematological Tumors	Phase 1
NX 2127	Nurix	BTK/IKZF1/IKZF3	B-cell Malignancies/Hematological Tumors	Phase 1
DG01	Degen Biomedical	GSPT1	Metastatic castration-resistant prostate cancer	Phase 1
AUTX-703	Auron	KAT2A/B	Myelodysplastic Syndromes	Phase 1

By mid-2025, three PROTACs, vepdegestrant (ARV-471), catadegbrutinib (BGB-16673), and gridegalutamide (BMS-986365), had advanced to Phase 3 in the United States. Notably, vepdegestrant, supported by Phase 3 data in ER^+^/HER2^−^ advanced breast cancer, has been submitted for FDA approval, positioning it as the potential first marketed PROTAC. This trajectory, from first clinical entry in 2019 to regulatory submission in just 6 years, exemplifies the technology’s rapid maturation and industry-wide validation. In summary, PROTAC technology has evolved from a conceptual degradation strategy into a clinically validated platform with tangible commercial prospects, and the impending approval of its first-in-class agents heralds its transition into an industrialization phase.

### The core challenges of PROTAC’s clinical transformation

Although PROTAC technology has made great progress in recent years, with vepdegestran and other candidate drugs entering phase 3 trials and submitting NDA, this revolutionary technology still faces serious challenges on the road to commercialisation [29, 60].

#### Unfavorable physicochemical properties

PROTACs are heterobifunctional molecules composed of two protein ligands connected by a linker, resulting in molecular weights that substantially exceed those of conventional small-molecule drugs, typically approaching ~1000 Da and thus violating Lipinski’s Rule of Five [61–64]. While this architecture is essential for stabilizing the POI-PROTAC-E3 ternary complex, it introduces significant transport barriers. Compounding this issue, PROTACs often exhibit high lipophilicity (LogP > 5) and extremely poor aqueous solubility, creating a constellation of interrelated physicochemical liabilities [4, 65]. These attributes collectively compromise cellular permeability (Fig. 1c), as excessive membrane affinity hinders efficient transmembrane passage to reach intracellular targets, directly impairing degradation activity even when the warhead is optimized. Importantly, although several PROTACs have advanced into clinical evaluation, their exact plasma membrane permeation mechanisms remain incompletely defined. In most cases, cellular entry is still believed to rely primarily on the intrinsic physicochemical properties of the molecules themselves, rather than on any well-established active transport pathway. Parameters such as polarity, molecular flexibility, lipophilicity, and intramolecular hydrogen bonding may collectively modulate transmembrane behavior and thereby influence intracellular exposure. Nevertheless, mechanistic studies on how individual clinical-stage PROTACs cross the plasma membrane remain limited, highlighting an important area for future investigation [31, 65–67]. Furthermore, poor solubility poses significant hurdles for formulation development, frequently resulting in low oral bioavailability and necessitating complex nanocarriers or specialized formulations that increase cost and limit administration routes [68–71]. This unfavorable physicochemical profile also culminates in non-specific tissue distribution, where high LogP promotes avid binding to biological membranes and plasma proteins. Such non-specific interactions reduce the concentration of free drug, diminish effective accumulation in target tissues, and can accelerate metabolic clearance [65, 72–74]. Consequently, these properties are now widely recognized as a primary impediment to the clinical translation of PROTACs, particularly restricting their therapeutic potential in oncology and neurodegenerative diseases [75].

#### Targeting selectivity and off-target effects

The event-driven pharmacology of PROTACs, while offering unique advantages, introduces distinct challenges regarding target selectivity and off-target effects [76]. These challenges stem primarily from three interconnected factors: the restricted tissue distribution of E3 ligases, the potential amplification of ligand inherent off-target risks, and the hook effect. These issues are frequently compounded by the intrinsic physicochemical limitations of PROTACs, such as poor membrane permeability, further complicating their delivery and efficacy.

The expression patterns of E3 ligases exhibit significant cell and tissue specificity, which fundamentally dictates the operational scope of a PROTAC [77–80]. Effective degradation necessitates that the target cell type specifically expresses the cognate E3 ligase to facilitate the formation of a stable E3-PROTAC-POI ternary complex [17, 81–83]. This requirement can confine PROTAC activity to specific cellular or microenvironmental contexts. Accordingly, for clinical-stage PROTACs, selectivity is more appropriately understood primarily at the levels of target engagement and degradation profile, whereas strict cell-type selectivity is not generally considered an intrinsic property. Rather, any apparent cellular selectivity is likely determined jointly by factors such as target protein expression, E3 ligase distribution, tumor microenvironment (TME) features, and delivery strategy. The stability of the protein-protein interactions within this ternary complex is a critical determinant of this selectivity; unstable complexes may allow the target protein to evade degradation, thereby compromising the consistency of the therapeutic effect [84].

Concurrently, the inherent propensity of the POI or E3-targeting ligands for non-target binding can be amplified within a PROTAC molecule, leading to the unintended degradation of off-target proteins [69]. Even with optimized ligand design, the formation of weak or unstable ternary complexes can erode selectivity. For instance, certain PROTACs have been shown to bind to over 50 kinases in vitro but effectively degrade only a small subset, underscoring the potential for off-target toxicity and the challenges in achieving high selectivity (Fig. 1c) [76, 85–90]. Resistance mechanisms also pose a significant hurdle [91–93]. While PROTACs can theoretically overcome resistance associated with conventional inhibitors, their efficacy in practice may be compromised by tumor immune evasion or the downregulation of E3 ligase expression. Mutations in the target protein that prevent POI ligand binding, or reduced E3 ligase levels that impair ubiquitination, can both abrogate ternary complex formation and subsequent degradation [94–100]. Furthermore, competition from endogenous substrates of the E3 ligase can also hinder the degradation of the intended neosubstrate [101].

Moreover, after systemic administration, PROTACs may undergo substantial dilution and loss of effective exposure before reaching target cells due to circulation, tissue distribution, protein binding, metabolic clearance, and nonspecific uptake [4], further underscoring the need for efficient delivery systems. Finally, the “hook effect” represents a unique, concentration-dependent phenomenon for PROTACs. At supraoptimal concentrations, PROTAC molecules increasingly form non-productive binary complexes (PROTAC-POI or PROTAC-E3) rather than the desired ternary complex [102, 103]. This self-inhibition at high doses diminishes degradation efficiency, confounds the dose-response relationship, and complicates efficacy predictions, while potentially elevating the risk of systemic side effects (Fig. 1c) [31, 104]. It also remains uncertain whether such non-productive binary complexes could, in certain contexts, associate with membrane-surface proteins and facilitate clustering-mediated endocytic uptake. Although this possibility cannot be excluded in principle, current experimental evidence remains insufficient, and further mechanistic investigation is needed.

#### Complex in vivo pharmacokinetic and pharmacodynamic hurdles

The physicochemical properties and selectivity limitations of PROTACs manifest as highly complex pharmacokinetic (PK) and pharmacodynamic (PD) behaviors in vivo, leading to a nonlinear relationship between drug exposure and therapeutic effect that significantly undermines clinical predictability [65, 105]. Poor oral bioavailability remains a primary barrier. Owing to the unfavorable physicochemical features described above (Fig. 1c), PROTACs typically exhibit minimal absorption upon oral administration. Their pronounced hydrophobicity and nonspecific tissue distribution further compound these issues, particularly restricting their application in diseases requiring systemic intervention, such as cancer [106]. ARV-110, for instance, advanced to Phase 2 trials at a high oral dose of 420 mg once daily, consistent with its limited rat bioavailability of 23.8% [107]. Beyond necessitating higher doses and increasing patient burden, low bioavailability raises the risk of the “hook effect”, where excessive concentrations promote the formation of non-productive binary complexes over functional tertiary complexes, thereby diminishing degradation efficiency.

Secondly, heterogeneous tissue distribution and low selectivity contribute to variable in vivo exposure. PROTACs frequently fail to accumulate effectively in target tissues or subcellular compartments, while off-target deposition in organs such as the liver or adipose tissue may potentiate toxicity [10]. Studies employing functionalized PROTACs to enhance tumor targeting reveal that peripheral distribution not only amplifies the risk of adverse effects but also diminishes the effective dose reaching the intended site of action [108]. Additionally, poor blood-brain barrier (BBB) penetration severely constrains central nervous system (CNS) applications [109–111]; the high molecular weight and polar nature of PROTACs impede CNS entry, necessitating the development of specialized delivery systems [112].

Rapid systemic clearance and metabolic instability present further obstacles [113]. For example, the Crews group reported the BTK degrader MT-802 as highly potent in vitro (low nanomolar DC₅₀), yet its high in vivo clearance and short half-life precluded further advancement [114]. In broader terms, PROTACs may undergo swift metabolic degradation or rapid elimination via renal or hepatobiliary routes, leading to inadequate systemic exposure, truncated duration of action, and a requirement for frequent dosing to sustain degradation activity [115, 116].

More critically, the PK/PD relationship of PROTACs is highly nonlinear and inherently difficult to predict [88]. This complexity arises from multifactorial interactions, catalytic degradation kinetics, the hook effect, and tissue-specific E3 ligase expression, that decouple plasma concentration from pharmacological effect. Indeed, high exposure may sometimes attenuate efficacy via the hook effect. Such intricate PK/PD behavior substantially complicates dose optimization and represents a key bottleneck in translating PROTACs from bench to bedside [117–120].

Safety concerns are equally pressing. Several PROTAC candidates have recently been discontinued due to serious toxicity, underscoring field-wide challenges. FHD-609, a BRD9 degrader, elicited Grade 4 QTc prolongation and syncope in Phase 1 trials, resulting in a clinical hold by the FDA [121]. Similarly, CFT-8634 achieved robust BRD9 degradation but failed to demonstrate meaningful efficacy in heavily pretreated patients, prompting discontinuation [122]. These cases illustrate potential liabilities, including off-target degradation of essential cardiac proteins, unintended proteolysis of non-target substrates, and the delicate balance between efficacy and toxicity.

In summary, the core challenges impeding PROTAC clinical translation are intrinsic to their molecular architecture: unfavorable physicochemical properties engender poor permeability, formulation difficulties, and nonspecific biodistribution; target selectivity issues compromise degradation specificity and efficiency; and aberrant in vivo PK/PD behavior confounds therapeutic prediction and dosing regimens [35]. These obstacles are deeply interwoven, amplifying one another and collectively hindering clinical development. Emerging evidence underscores the imperative for integrated, multidisciplinary delivery strategies to overcome these barriers, thereby enhancing both the clinical utility and safety of PROTAC therapeutics.

## Innovative delivery strategies

DDS is the core field of modern pharmaceutical research, which aims to overcome the limitations of traditional drug administration methods by accurately regulating the spatio-temporal distribution, release rate and targeted delivery of drugs in organisms [123–126]. Complex delivery strategies can significantly improve the treatment index of drugs, enhance efficacy and reduce toxicity, thus acting as a key technical support for improving drug-like characteristics and expanding treatment windows [127–129]. Therefore, combining PROTAC technology with advanced drug delivery strategies to build an intelligent, efficient and targeted delivery system is an urgent need and inevitable direction to overcome the bottleneck of its in vivo application and give full play to its therapeutic potential [130].

In recent years, researchers have developed various innovative delivery platforms according to the unique requirements of PROTAC molecules. Through chemical modification, carrier encapsulation or biological conjuncture, these platforms give PROTAC molecules key characteristics, such as controlled release, targeted delivery, improved pharmacokinetics and reduced toxicity [131–135]. This section aims to systematically review and classify the currently reported PROTAC-based innovative delivery strategies, comprehensively outline the research progress in this field, and provide insights for the future design of more efficient and secure PROTAC delivery systems.

### Prodrugs

**Fig. 2:**
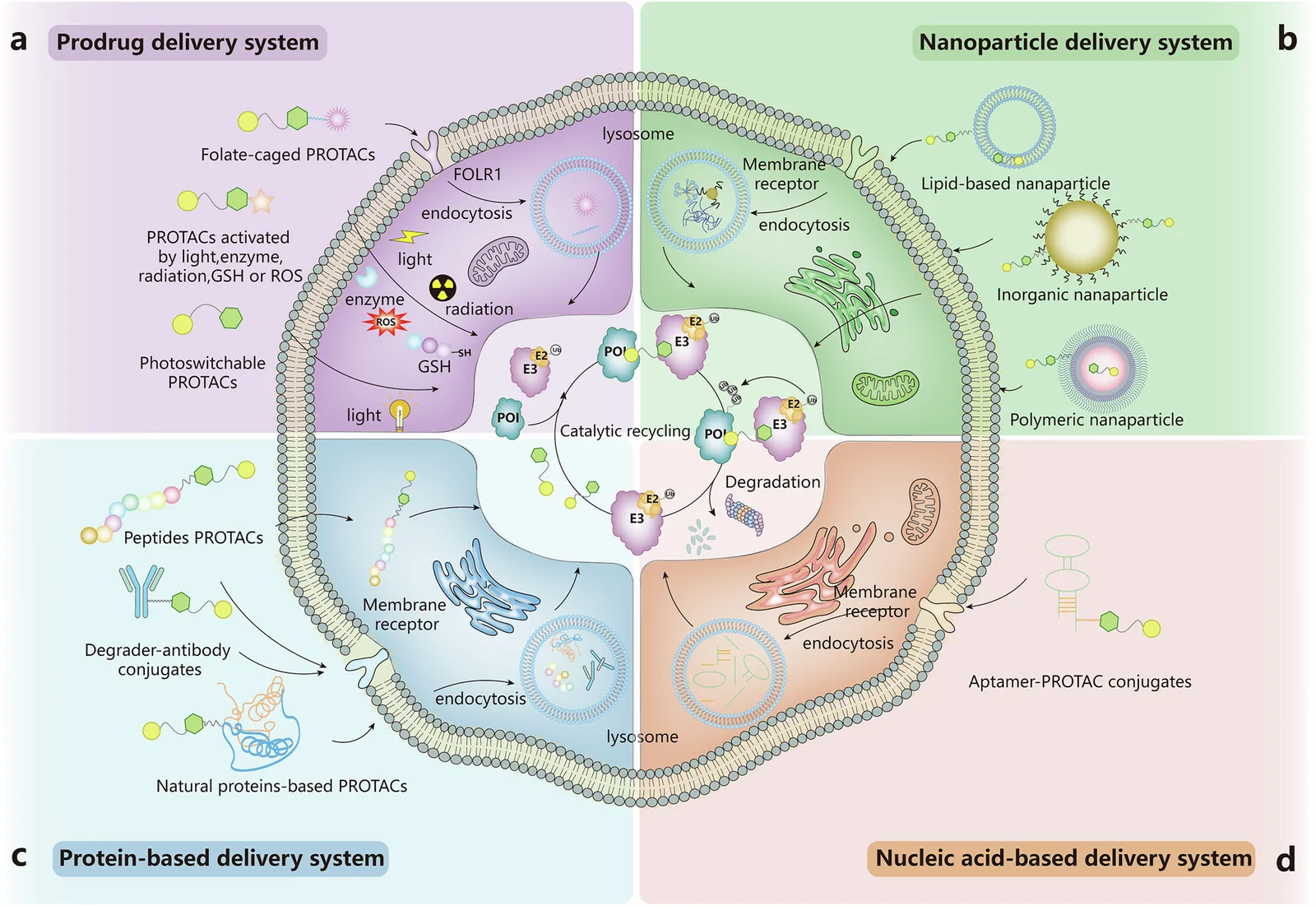
Schematic diagrams of PROTAC-induced protein degradation mediated by various delivery platforms. These platforms improve the delivery efficiency and therapeutic potential of PROTACs through different strategies. **a** Various types of PRO-PROTACs have become a promising strategy to release active PROTACs specifically in tissues. **b** Various nano-PROTACs based on lipids, polymers, inorganic materials and other substrates significantly optimise the delivery efficiency and treatment potential of traditional PROTACs through a variety of technical methods, so that PROTACs can be accurately released in the tissue. **c** The PROTACs delivery system based on natural protein peptide antibodies benefits from the targeted identification ability of proteins, so that drugs can be transported to specific sites. **d** As an artificially synthesised single-stranded DNA or RNA oligonucleotide, Aptamers can be folded into a specific three-dimensional structure. This enables them to combine with target molecules with high affinity and selectivity, making them ideal components for the construction of targeted PROTAC delivery systems.

In order to meet the challenges posed by the inherent unfavorable physicochemical and pharmacokinetic characteristics of PROTAC molecules, PROTAC (PRO-PROTAC) based on prodrugs has become a promising strategy in recent years (Fig. 2a). This method aims to achieve the specific release of active PROTAC in tumor tissue while minimizing the toxicity of extra-target tissue [136–139].

#### Photo-controllable PROTACs

The precise spatiotemporal control afforded by light has been extensively leveraged in biological applications, leading to the development of two principal strategies for designing photo-controllable PROTACs: the incorporation of photocaging groups and the integration of photoswitches [140, 141].

**Fig. 3:**
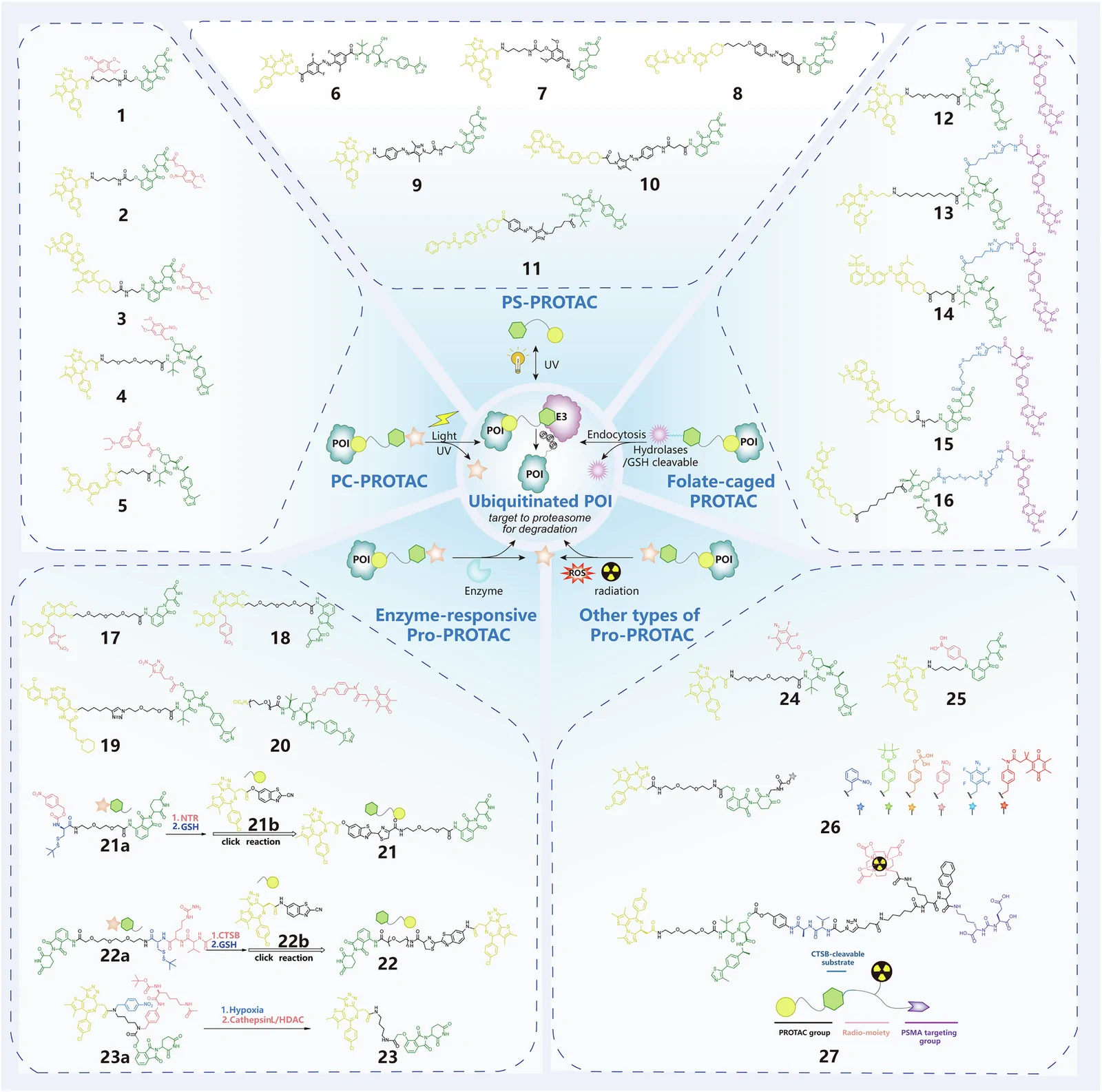
The chemical structure of representative molecules from five categories that can activate the PRO-PROTAC platform. Photocaged PROTACs (PC-PROTACs), covalent modification with the optical instability group, used for optical control activation; photoswitch PROTACs (PS-PROTACs), incorporating azobenzene photoswitch into the linker, used for reversible, photo-dependent adjustment of degradation activity; folate-caged PROTACs, activated by FOLR1-mediated endothelial cell dissolution and subsequent hydrolysis; enzyme response Pro-PROTACs, using enzyme-moveable mask groups for selective activation in a specific microenvironment; and other types of Pro-PROTACs, through cage opening and the self-decomposition and lysis mechanism realises part-specific activation and traceless release. These systems jointly realise the spatial and temporal control of PROTAC activity and targeted protein degradation.

Photocaged PROTACs (PC-PROTACs) are rendered inactive through the installation of photosensitive caging moieties, which are cleaved upon light irradiation to release the active degrader. This approach was pioneered by Xue et al. in 2019, who introduced a 4,5-dimethoxy-2-nitrobenzyl (DMNB) group into the BRD4-targeting PROTAC dBET1, yielding the first photocaged PROTAC (pc-PROTAC **1**) (Fig. 3), which remained inert in the dark but effectively degraded BRD4 upon light exposure in both cellular and zebrafish models [142]. Subsequently, Liu et al. [143] developed a prodrug strategy employing the nitroveratryloxycarbonyl (NVOC) group, capping the glutarimide nitrogen of pomalidomide to create Opto-dBET1 **2** (Fig. 3). The steric hindrance from NVOC reduced CRBN affinity, inactivating the molecule until UV-triggered photolysis restored potent, dose-dependent BRD3/4 degradation; the strategy’s generality was further confirmed with a photocaged ALK-targeting PROTAC, Opto-dALK **3** (Fig. 3). Extending this concept to the VHL E3 ligase system, Kounde et al. [144] masked the hydroxyl group of the VHL ligand with a DMNB group to generate photocaged pro-PROTAC **4** (Fig. 3), which released the active species upon irradiation to degrade BRD4 in HeLa cells. In a further expansion of chemical tools, Naro et al. [145] incorporated a diethylaminocoumarin (DEACM) cage into an estrogen-related receptor α (ERRα)-targeting PROTAC, producing photocaged PROTAC **5** (Fig. 3), whose light-activated degradation of ERRα underscored the applicability of the DEACM group for precise optical control.

In parallel, photoswitchable PROTACs (PS-PROTACs) achieve reversible modulation of degradation activity by integrating moieties, typically azobenzene derivatives, that undergo light-driven isomerization [146, 147]. Crews and colleagues [148] pioneered this strategy in 2019 by incorporating an ortho-tetrafluoroazobenzene switch into the linker of the VHL-based PROTAC ARV-771, creating photoPROTAC **6** (Fig. 3). The resultant *trans*-to-*cis* photoisomerization enabled spatiotemporal control over BRD4 degradation, establishing a core design paradigm. Focusing on the CRBN ligand system, Reynders et al. [149] embedded an azophenyl group into lenalidomide to construct PHOTAC-I-3 **7** (Fig. 3). In its active *trans* configuration under 390 nm light, it effectively reduced BRD4 and c-MYC levels, while 500 nm light restored the inactive state; notably, this work introduced wavelength-mediated control of active PROTAC concentration to predictably avoid the “hook effect”. Jin et al. [150] applied this technology to oncoproteins such as ABL/BCR-ABL, developing Azo-PROTAC **8** (Fig. 3), whose *trans* isomer degraded the target in K562 cells, with visible light-induced *cis* isomerization reversing the effect and enabling a reversible knockdown cycle in a tumor model. Expanding the chemical diversity of photoswitches, Zhang’s team [151] employed an arylazopyrazole moiety to create AP-PROTAC-1 **9** (Fig. 3) (targeting BRD2/4) and AP-PROTAC-2 **10** (Fig. 3) (targeting kinases FAK, Aurora-A, and TBK1), which exhibited improved photostationary state distribution and switching dynamics, with the trans isomers being active and *cis* isomers inactive. Demonstrating significant in vivo potential, Cheng’s team [152] designed a NAMPT-targeted PS-PROTAC **11** (Fig. 3) that innovatively utilized an activator as the target ligand. This azobenzene-based design allowed reverse photoregulation of NAMPT activity and downstream NAD^+^ levels, crucially avoiding the off-target toxicity in the dark state that plagues inhibitor-based PS-PROTACs and thereby laying a foundation for clinical applications.

Despite these advances, the translational applicability of photo-controllable PROTACs is still constrained by the limited tissue penetration of light and potential phototoxicity. Accordingly, although photocaged PROTACs have shown good controllability in model organisms such as zebrafish, broad and safe activation in humans remains difficult. However, they may still be promising for superficial tissues, localized lesions, or applications supported by advanced light-delivery strategies.

#### Folate-caged PROTACs

The tumor-specific overexpression of folate receptor alpha (FOLR1/FRα) on malignant cells, coupled with its limited presence in normal tissues, establishes it as a strategic conduit for the targeted delivery of anticancer therapeutics. This biological foundation has been exploited to develop prodrug forms of PROTACs (Pro-PROTACs), aimed at mitigating the systemic toxicity associated with off-target PROTAC activity. Liu et al. [10] pioneered this approach with a FOLR1-targeted delivery system, constructing folate-caged PROTAC prodrugs such as folate-ARV-771 **12** (Fig. 3), folate-MS432 **13** (Fig. 3), and folate-MS99 **14** (Fig. 3). These conjugates leverage FOLR1-mediated endocytosis for selective entry into cancer cells, enabling the specific degradation of BRD, MEK1/2, and ALK fusion proteins without affecting non-malignant cells. However, efficient endosomal escape and intracellular release of the active PROTAC remain important determinants of the ultimate degradation efficacy. To refine controllability, Chen et al. [153] introduced a reduction-responsive folate-PROTAC (FA-S2-MS4048, **15**) (Fig. 3) by linking folate to a pomalidomide derivative via a disulphide bond. This design harnesses the elevated intracellular glutathione (GSH) levels in tumor cells to cleave the linker, releasing the active PROTAC for FOLR1-dependent ALK degradation. Addressing the concurrent challenges of poor solubility and uncontrolled biodistribution, Ma’s team [154] engineered a folate-PEG-PROTAC micelle (MPRO) system. This platform incorporates folate for targeting and polyethylene glycol (PEG) chains for enhanced hydrophilicity, with a disulphide bond tethering an EGFR-targeted PROTAC to form compounds **16** (Fig. 3). These constructs self-assemble into nanoparticles that exhibit tumor-specific targeting and microenvironment-responsive release, thereby improving solubility and enabling precise intracellular activation.

#### Enzyme-responsive Pro-PROTACs

Compared to normal tissues, tumor cells exhibit distinct pathophysiological traits, including rapid proliferation, metabolic dysregulation, and heightened invasive potential. These phenotypes are commonly accompanied by elevated reductive stress, hypoxia within the TME, and the overexpression of various functional enzymes [155–158]. Such discrepancies provide a rational basis for designing tumor-selective, stimulus-activatable prodrugs of proteolysis-targeting chimeras (Pro-PROTACs). Among these, enzyme-responsive strategies have emerged as a pivotal avenue for achieving precise degradation specificity. By exploiting tumor-associated enzymes, such as dehydrogenases, hydrolases, and cathepsins, PROTAC activity can be gated with enhanced tissue selectivity and minimized off-target effects [159, 160].

Nitroreductase (NTR), which shows heightened activity under hypoxic conditions, has drawn particular interest. Nitroimidazole, a classic NTR substrate, undergoes enzyme-mediated cleavage and has been leveraged to construct hypoxia/NTR-activatable Pro-PROTACs [161, 162]. In pioneering work, Cheng et al. incorporated hypoxia-labile groups e.g., (1-methyl-2-nitro-1H-imidazol-5-yl)methyl or 4-nitrobenzyl into an EGFRdel19-targeting PROTAC, yielding ha-PROTACs **17** and **18** (Fig. 3) [163]. These conjugates exhibited markedly enhanced degradation of EGFRdel19 under hypoxic versus normoxic conditions, establishing the feasibility of hypoxia-triggered PROTAC activation. Shi et al. further advanced this concept by installing a nitroimidazole moiety on the VHL ligand, generating Pro-PROTAC **19** (Fig. 3) [160]. This design enables NTR-mediated release of active PROTAC and effective EGFR degradation. Notably, placing the responsive unit on the E3 ligase ligand side offers broader generalizability compared to earlier ha-PROTAC designs.

Subsequent innovations have integrated multi-stimuli responsiveness to refine selectivity. In 2022, Do et al. introduced ENCTAC, a dual-responsive system activated by NTR and glutathione (GSH) for BRD4 degradation [164]. Under hypoxia, NTR triggers nitro group departure from pomalidomide; the ensuing 1,2-aminothiol then undergoes click conjugation with cyanobenzothiazole (CBT)-modified JQ1 in the GSH-rich TME, forming active PROTAC J252 **21** (Fig. 3). Similarly, Sun et al. developed a cathepsin B (CTSB)/GSH dual-responsive clickable Pro-PROTAC, utilizing an Ac-Val-Cit-Cys(StBu) peptide on pomalidomide [108]. CTSB-mediated cleavage followed by GSH-dependent thiol exposure enables click ligation with CBT-labeled JQ1, yielding active PROTAC **22** (Fig. 3). These clickable platforms circumvent the synthetic challenges of conventional PROTACs and demonstrate superior tumor penetration due to their lower molecular weight.

Pursuing even stricter spatiotemporal control, Dutta et al. [165] proposed the “Dual-Activation Gated” Pro-PROTAC concept, exemplified by DAO-dBET1 **23a** (Fig. 3). This molecule requires both hypoxia and cathepsin L activity for full activation, thereby integrating two TME-specific cues to achieve enhanced safety windows and precise BRD4 degradation. Beyond NTR and cathepsins, other tumor-associated enzymes have also been exploited. Liang et al. [166] reported an NAD(P)H:quinone oxidoreductase 1 (NQO1)-responsive Pro-PROTAC **20** (Fig. 3), which remains inert until activated by NQO1 overexpression in cancer cells. Collectively, these enzyme-reactive Pro-PROTAC designs underscore the versatility of leveraging endogenous tumor characteristics to govern PROTAC function, offering a robust framework for advancing TPD therapeutics.

#### Other types of Pro-PROTACs

Radiotherapy (RT), which employs high-energy X-rays to ablate cancer cells, remains a cornerstone of oncological intervention due to its precise spatial targeting and deep tissue penetration [167]. Harnessing this precision, Yang et al. pioneered a radiotherapy-triggered PROTAC (RT-PROTAC) by replacing the hydroxyl group of the VHL ligand with an X-ray-sensitive phenylazido group, creating prodrug **24** (Fig. 3). Upon X-ray irradiation, this caging group undergoes reduction to aniline, followed by 1,6-benzyl elimination and decarboxylation to liberate the active PROTAC, enabling targeted degradation of BRD4/2 [5]. Building on this concept, Zhang et al. integrated targeted radionuclide therapy (TRT) with PROTAC technology through the dual-functional molecule 177Lu-P-A **27** (Fig. 3), which combines a PSMA-targeting moiety for prostate cancer cell accumulation with a cathepsin B (CTSB)-cleavable linker. Following internalisation, CTSB cleavage releases the BRD4-targeted PROTAC, while the conjugated 177Lu delivers continuous TRT. Notably, the subsequent degradation of BRD4 enhances radiosensitivity and synergistically amplifies cytotoxic efficacy beyond that of monotherapy [168]. These studies exemplify two distinct activation strategies, direct X-ray-induced chemical release and combined TRT/enzyme-controlled liberation, advancing the spatiotemporal precision and therapeutic index of radiotherapy-triggered PROTACs.

Parallel to these external stimuli, endogenous cues such as reactive oxygen species (ROS), which are elevated in tumors relative to normal tissues, have been exploited for selective prodrug activation [169, 170]. Liu et al. developed a ROS-responsive PROTAC prodrug by introducing an aryl boric acid group as a cleavable caging moiety on the lenalidomide fragment. Exposure to H₂O₂ triggers conversion to a phenolic intermediate and subsequent 1,6-benzyl elimination, yielding active PROTAC **25** (Fig. 3) for TPD [171]. In a broader effort, An and colleagues systematically constructed a panel of stimuli-responsive PROTACs (sr-PROTACs), exemplified by ddBET1 **26** (Fig. 3), which integrates modular molecular switches responsive to ultraviolet light, hydrogen peroxide, phosphatase, nitroreductase, trans-cyclooctene, and NQO1. This design permits in situ activation within tumor cells, enhancing degradation specificity both in vitro and in vivo [172].

While prodrug strategies offer a compelling route to improve PROTAC selectivity by circumventing biological barriers and enabling tumor-specific activation [32], several challenges persist [173]. The pronounced spatial heterogeneity of the solid TME can lead to variable activation efficiencies across different lesion regions, potentially compromising therapeutic thoroughness (Table 2) [174, 175]. In addition, caging-group installation and prodrug activation kinetics require careful optimization to avoid premature cleavage, over-stabilization, or incomplete activation [10, 176]. Additionally, the metabolic byproducts released upon activation could introduce unforeseen safety liabilities. Even upon successful tumor targeting, efficient intracellular prodrug conversion and formation of functional ternary complexes can be impeded by the complex endomembrane environment, thereby attenuating actual degradation efficacy [177–180]. These factors, together with the multi-parameter optimization of activity, stability, and activation responsiveness, increase design and synthetic complexity and may hinder clinical translation [178]. Thus, while the prodrug approach holds substantial promise for broadening PROTAC therapeutic applicability, overcoming its inherent dependence on heterogeneous microenvironmental conditions, delivery uncertainties, and synthetic complexities remains critical for advancing these technologies toward widespread clinical use.

**Table 2:** Key parameters of different delivery systems.

Delivery strategy	Advantages	Disadvantages	Key parameters	Applicable scenarios
Pro-PROTAC	High selectivity, improved pharmacokinetics (PK), reduced off-target toxicity.	Activation efficiency depends on tumor microenvironment heterogeneity; inactive metabolites may be generated; chemical synthesis may be complex.	*Activation conditions:* Tumor microenvironment-specific stimulation; *Tissue specificity:* Dependent on microenvironment heterogeneity, lacking absolute tissue selectivity; *PK optimization:* Significantly improved solubility and in vivo stability, reduced off-target toxicity.	Solid tumor therapy; high-activity PROTACs requiring avoidance of systemic toxicity.
LNPs	High drug loading capacity, easy functionalization, good biocompatibility.	Prone to oxidation and hydrolysis; may induce immune responses; difficult quality control in large-scale production.	*Particle size:* Typically 50–200 nm (dependent on preparation process); *Biocompatibility:* Phospholipid materials have good biocompatibility but may trigger complement activation; *Drug loading capacity:* High loading efficiency for lipophilic PROTACs; *Stability:* Susceptible to oxidation and hydrolysis, requiring formulation optimization.	Intravenous administration; targeting organs enriched in the mononuclear phagocyte system (MPS) such as liver and spleen.
Polymer Nanoparticles	Controlled release, surface multi-functional modification, long circulation.	Low drug loading capacity; polymer metabolites need evaluation; batch-to-batch variation.	*Particle size:* Typically 100–300 nm; *Degradation properties:* Polymers such as PLGA can control degradation rate via monomer ratio to achieve sustained release; *Biocompatibility:* Polymer metabolites need evaluation, generally good safety; *Drug loading capacity:* Low for encapsulated type.	Dosing scenarios requiring long-acting sustained release; targeted tumor therapy.
Inorganic Nanoparticles	High stability, unique properties, precise structural controllability.	Poor biodegradability; long-term in vivo safety concerns; difficult metabolic clearance.	*Particle size:* Mesoporous silica is typically 50–200 nm; gold nanoparticles can be as small as several nanometers; *Stability:* Excellent chemical/physical stability, suitable for multimodal functional integration; *Biodegradability:* Inorganic materials degrade slowly, long-term retention may induce chronic inflammation.	Multimodal combination therapy (e.g., chemotherapy-PROTAC); theranostics (integration of diagnosis and therapy).
APCs	Precise targeting, prolonged half-life, reduced systemic exposure.	Large molecular weight; poor penetration in solid tumors; extremely high production cost; complex linker chemistry and release mechanism.	*Immunogenicity:* Antibodies may induce immune responses, requiring humanization to reduce immunogenicity; *Molecular weight:* > 150 kDa, weak penetration ability in solid tumors; *Linker design:* Need to balance circulatory stability and tumor microenvironment responsiveness; *Targeting specificity:* Dependent on high specificity of antibody-antigen binding, strong tissue selectivity.	Hematological tumors or solid tumors with high expression of specific antigens.
Peptide-Based PROTACs	High cell penetration efficiency; targeting peptides enable tissue/cell specificity; good biocompatibility; low immunogenicity; easy chemical synthesis and modification.	Peptides are susceptible to protease degradation; poor serum stability; potential off-target penetration; conjugation process may affect PROTAC activity.	*Activation conditions:* Mostly passive penetration or receptor-mediated endocytosis; some can respond to the tumor microenvironment; *Particle size:* Conjugates are usually at the small-molecule scale ( < 10 nm); assemblies are 10–100 nm; *Immunogenicity:* Natural peptides have extremely low immunogenicity, far lower than antibodies; Stability: Susceptible to serum protease degradation; *Targeting specificity:* Dependent on the receptor-binding specificity of peptides.	Targeted delivery of PROTACs that are difficult to penetrate solid tumors; intracellular target PROTAC delivery; repeated dosing scenarios requiring low immunogenicity.
DACs	Small molecular weight, good penetrability, easy chemical synthesis and modification, low immunogenicity.	Poor serum stability; susceptible to nuclease degradation; rapid renal clearance.	*Immunogenicity:* Extremely low immunogenicity, far superior to antibodies; *Serum stability:* Susceptible to nuclease degradation; *Molecular weight:* < 50 kDa, high penetration efficiency in solid tumors; ***Affinity:*** Affinity is usually weaker than that of antibodies.	Solid tumor therapy requiring rapid penetration; proof-of-concept (POC) as research tools.

### Nanomaterial-based delivery systems

The breakthrough of nanotechnology has greatly promoted translational medicine. Nanoparticles have been widely used as drug delivery carriers in PROTAC technology. Current research reports a variety of nanoscale. formulas, including lipid-based, polymer-based and inorganic material-based systems (Fig. 2b) [181–184], aiming to optimize the delivery efficiency and therapeutic potential of PROTAC. Importantly, the timing and location of PROTAC release in these nanomaterial-based delivery systems depend strongly on carrier composition and trigger mechanism. In general, payload release occurs in the TME or after cellular internalization within endosomal/lysosomal compartments, and in some systems may further proceed following endosomal escape, thereby determining the cytosolic availability of the active degrader [184–186].

#### Lipid-based nanoparticles

**Fig. 4:**
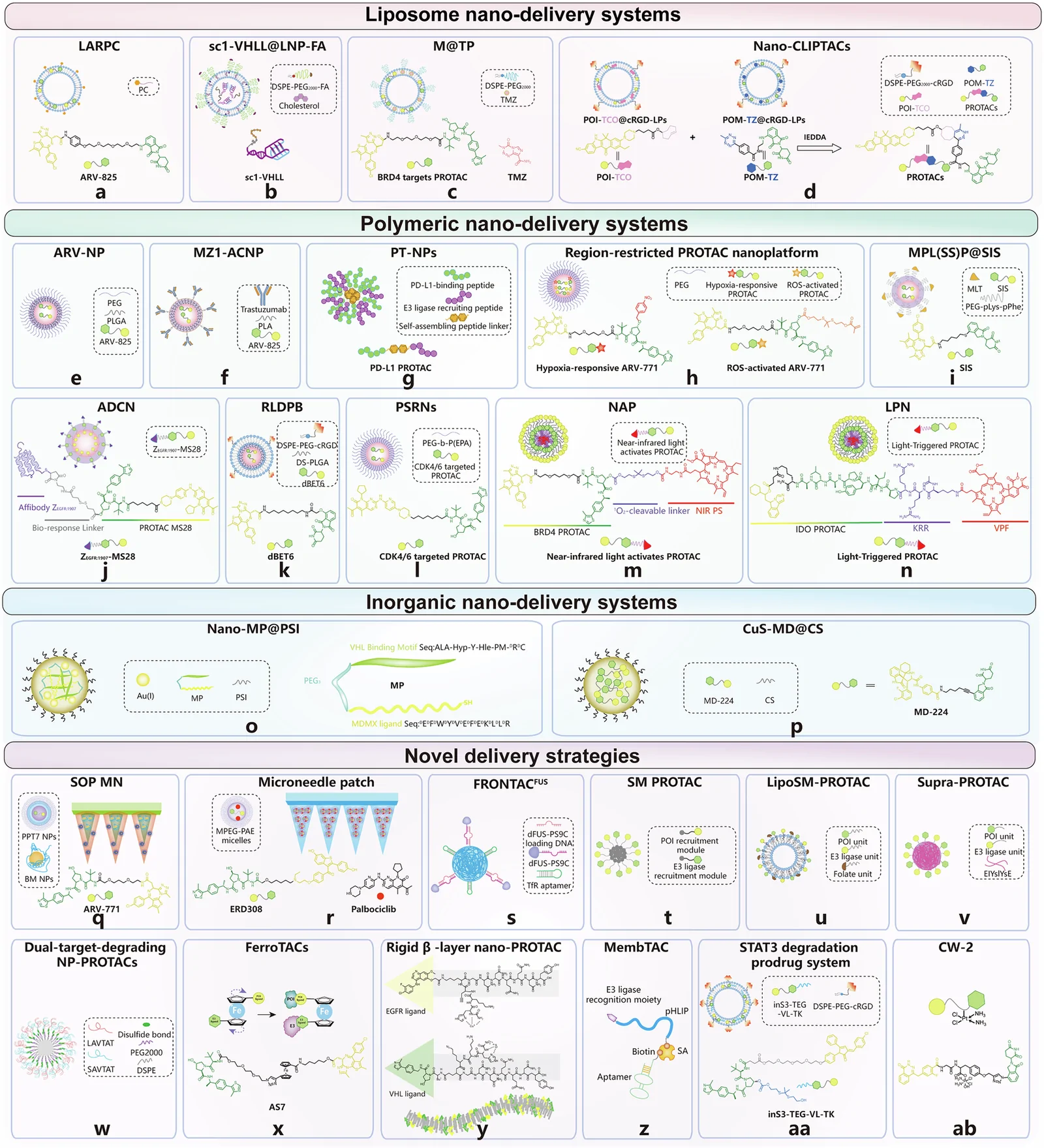
The chemical composition of various PROTAC delivery platforms. These systems aim to solve the delivery challenges of PROTAC through different strategies. **a**–**d** Lipid nanoparticles use their membrane-imitation structure and biocompatibility to achieve stable tumour accumulation and controlled intracellular release. **e**–**n** Polymer nanoparticles achieve flexible PROTAC loading through encapsulation, surface modification or covalent covalent conjugation, enhancing tumour targeting accumulation and adjustable release dynamics. **o**, **p** Inorganic nanoparticles use their inherent stability and biocompatibility to improve the pharmacokinetic characteristics of PROTAC. **q**–**ab** The new and multidisciplinary delivery system combines advanced non-nanoparticles and hybrid designs to achieve precise, efficient and space-time control PROTAC conveying. These platforms together represent multifunctional and extensive toolkits to optimise PROTAC-based treatments.

Lipid nanoparticles (LNPs) and liposomes have emerged as versatile delivery platforms for PROTACs, capitalizing on their architecturally designable vesicle structures composed of phospholipid bilayers encapsulating a hydrophilic core. This unique architecture facilitates the co-delivery of both hydrophobic and hydrophilic therapeutic agents, offering key advantages such as formulation simplicity, excellent biocompatibility, high drug loading, and tunable physicochemical properties [185]. Recent innovations in lipid-based PROTAC delivery are exemplified by Fu and colleagues, who developed PEGylated nanoliposomes (**LARPC**) (Fig. 4a) for the concurrent delivery of the BRD4-targeting PROTAC ARV-825 and a protein kinase C inhibitor. This system potently suppressed both angiogenesis and vasculogenic mimicry in vemurafenib-resistant melanoma models [186]. Similarly, Peng et al. constructed **sc1-VHLL@LNP-FA** (Fig. 4b), an ionizable LNP platform encapsulating a PROTAC targeting fragile X mental retardation protein (FMRP), enabling efficient tumor-targeted delivery and FMRP degradation, which synergized with immune checkpoint inhibitors to enhance anti-tumor immunity [187]. The utility of LNPs has been further expanded by Andrew Tsourkas’ team, who employed them to deliver protein-based bifunctional degraders (bioPROTACs), thereby overcoming the membrane permeability limitations of small-molecule PROTACs and achieving TPD within subcellular compartments like mitochondria and the nucleus [188].

Beyond conventional lipid formulations, biomimetic design strategies have markedly enhanced targeting precision and broadened the translational potential of nanocarriers. These principles have been successfully integrated into diverse platforms, including liposomes, polymers, and inorganic nanoparticles [189–192]. For instance, Xu et al. engineered a hybrid liposome (**M@TP**) (Fig. 4c) camouflaged with membranes derived from drug-resistant glioma cells, enabling the targeted co-delivery of a BRD4-targeting PROTAC and temozolomide. This biomimetic approach facilitated BBB penetration and specific homing to glioblastoma, resulting in pronounced growth inhibition of temozolomide-resistant tumors [193]. In the context of neurodegenerative diseases, Gong and colleagues harnessed neurotransmitter-derived lipid (NT-lipid) nanoparticles to encapsulate a tau-targeting PROTAC, yielding a nanodrug (NPD) with high encapsulation efficiency, robust serum stability, and superior BBB permeability. NPD effectively induced tau clearance in both cultured neurons and the brains of Alzheimer’s disease model mice [41]. Addressing the need for tumor-specific activation, Xie’s team pioneered the “**Nano-CLIPTACs**” (Fig. 4d) strategy, wherein two PROTAC precursor molecules were separately encapsulated in cRGD-functionalized nanoliposomes. Upon binding to αvβ3 integrin on tumor cells, the precursors underwent an in situ inverse electron-demand Diels–Alder (IEDDA) reaction within the TME to assemble the functional PROTAC. This approach significantly amplified anti-tumor efficacy while minimizing systemic toxicity, presenting a novel paradigm for precision cancer therapy [194].

#### Polymeric nanoparticles

Polymer nanoparticles have emerged as a versatile delivery platform for PROTACs, offering enhanced tumor-specific accumulation and controlled release through physical encapsulation, surface modification, or chemical conjugation [195]. Early work by Saraswat et al. demonstrated this concept using PLGA-PEG nanoparticles loaded with ARV-825 (**ARV-NP**) (Fig. 4e), which leveraged the enhanced permeability and retention (EPR) effect for passive tumor targeting and exhibited potent in vitro anti-tumor activity [196]. Building on this, the Cimas team developed actively targeted systems by encapsulating the BRD4 degrader MZ1 in polylactic acid (PLA) nanoparticles and conjugating trastuzumab (Tz) to the surface, conferring specific uptake in HER2-overexpressing breast cancer cells. This antibody-mediated delivery, combined with the controlled release properties of the nanoparticle, resulted in significantly enhanced cytotoxicity of **MZ1-ACNPs** (Fig. 4f) compared to free MZ1 in HER2-positive cell lines [197].

The field has since advanced toward intelligently responsive systems that enable precise spatiotemporal control over PROTAC activity. Moon et al. designed self-assembling peptide-based PROTAC nanoparticles **(PT-NPs**) (Fig. 4g) incorporating a PD-L1 binding peptide, a self-assembly linker, and an E3 ligase ligand. Upon receptor-mediated endocytosis and lysosomal degradation, these nanoparticles release free PROTAC, leading to cytoplasmic PD-L1 degradation via the UPS and offering a new strategy for cancer immunotherapy [198]. Parallel to this, Gao et al. developed a **regionally limited PROTAC nanoplatform** (Fig. 4h) integrating both ROS-sensitive and hypoxia-responsive elements, allowing precise temporal and spatial regulation of BRD4 expression [199]. Exploiting the TME further, Guo’s team created glutathione (GSH)-reactive nanocapsules (**MPL(SS)P@SIS**) (Fig. 4i) using biocompatible PEG-pLys-pPhe polymers loaded with the BRD4 degrader SIS. Surface modification with 1-methyl-*L*-tryptophan (MLT) enabled LAT1 transporter-mediated BBB penetration and glioblastoma accumulation, with tumor-specific PROTAC release enhancing radiosensitisation while minimizing systemic toxicity [200].

Functionalised self-assembly strategies have also yielded major breakthroughs. Yan’s group constructed an amphiphilic conjugate (ZEGFR:1907-MS28) by linking a hydrophilic EGFR-targeting moiety to a hydrophobic PROTAC (MS28) via a disulphide bond, which spontaneously assembles into nanodrugs (**ADCNs**) (Fig. 4j) under physiological conditions. Tumoural GSH triggers PROTAC release, leading to effective cyclin D1 degradation and tumor growth inhibition [201]. Guan et al. integrated dual-targeting and bioresponsive features in a nano-PROTAC (**RLDPB**) (Fig. 4k) comprising a pH/GSH-responsive DS-PLGA polymer loaded with dBET6, cloaked in LLC lung cancer cell membranes, and decorated with cRGD for αvβ3 integrin targeting, significantly improving lung cancer therapeutic precision [202]. Similarly, Yang’s team designed ultra-pH-sensitive/enzyme-responsive nanoparticles (**PSRNs**) (Fig. 4l) that accumulate via EPR, dissociate in the acidic TME, and are activated by cathepsin B to release CDK4/6-targeting PROTACs with temporally controlled degradation profiles [203].

Exogenous stimulus-responsive systems have further expanded the dynamic regulation of PROTACs. Wang et al. reported near-infrared (NIR) photoactivatable nanoPROTAC (**NAP**) (Fig. 4m) self-assembled from a PROTAC, a singlet oxygen-cleavable linker, and an NIR photosensitiser. Upon tumor accumulation, NIR irradiation simultaneously triggers PROTAC release and photodynamic therapy (PDT), eliciting synergistic anti-tumor effects [204]. Choi’s group constructed light-triggered PROTAC nanocomponent **(LPN)** (Fig. 4n) incorporating a cathepsin B-cleavable peptide, the photosensitiser verteporfin, and an IDO-targeting PROTAC. Following EPR-mediated accumulation, enzymatic cleavage releases active PROTAC, which together with PDT induces immunogenic cell death, offering an innovative approach for colon cancer treatment [205].

#### Inorganic nanoparticles

Inorganic nanoparticles, with their tunable sizes, diverse geometries, and modifiable surfaces, have emerged as versatile platforms for PROTAC delivery, offering enhanced biocompatibility and the potential to overcome the pharmacokinetic limitations of these therapeutic agents. For instance, He and colleagues employed a nanoengineering strategy to transform a peptide-derived molecular glue (MP) targeting the oncoprotein MDMX into a supramolecular gold(I)-thiol-peptide complex (Nano-MP). This was further coated with a pH-responsive polymer (PSI) to create **Nano-MP@PSI** (Fig. 4o), a system that significantly improved cellular internalization, enabled glutathione-responsive release, and enhanced proteolytic stability, thereby boosting intracellular PROTAC delivery efficiency [206, 207]. In a complementary approach, Wang’s team developed **CuS-MD@CS** (Fig. 4p), an inorganic-organic nanoplatform utilizing copper sulfide nanoparticles as a core for the MD-224 PROTAC, with a chondroitin sulfate coating to improve tumor targeting and coordination-based delivery precision [208].

While lipid-based nanoparticles (LNPs) have garnered significant clinical traction, evidenced by their successful deployment in mRNA vaccines and offering promise for challenging therapeutics like PROTACs (Table 2) [209, 210], the path to clinical translation for nanocarrier-based PROTAC delivery is fraught with hurdles. The inherent physicochemical duality of PROTACs, possessing both hydrophilic and hydrophobic regions, complicates their efficient loading and stable encapsulation. Furthermore, the long-term in vivo fate of nanocarrier materials, including potential accumulation and immunogenicity, remains inadequately characterized [43]. Even upon reaching the tumor site, the nanocarrier’s size and surface properties can impede deep tissue penetration, while stimulus-responsive release mechanisms may prove unreliable in the complex in vivo milieu, leading to suboptimal or uncontrolled drug release [211]. For nanocarrier systems that enter cells via endocytosis, endosomal escape is a critical prerequisite for productive cytosolic delivery of PROTACs. Without efficient escape, released PROTACs may remain trapped within endosomal/lysosomal compartments, thereby limiting their access to cytosolic or nuclear targets and E3 ligases and ultimately attenuating degradation efficacy. In currently reported nanodelivery systems, this step is often facilitated by ionizable lipid formulations, pH-responsive materials, enzyme-triggered release, or receptor-mediated intracellular trafficking [187, 198, 200, 203, 205–207]. Nevertheless, insufficient endosomal escape efficiency remains one of the major bottlenecks in nanocarrier-based PROTAC delivery [211]. Compounding these biological challenges are significant manufacturing and quality control obstacles; scaling up production from the laboratory to a clinical setting requires maintaining stringent control over sensitive processes, where minor fluctuations can result in batch-to-batch variability and prohibitive costs [212]. Despite these considerable challenges, nanoparticle-based delivery systems continue to hold substantial promise as a pivotal enabling technology for advancing PROTACs toward clinical application.

### Protein-based delivery systems

Natural biological macromolecules, especially proteins, have become an attractive platform for drug delivery in the body because of their favorable biocompatibility and unique molecular structure [213]. The targeted identification ability granted by its three-level structure makes it possible to transport specific parts of therapeutic agents. Therefore, the protein-based PROTAC system (Fig. 2c) represents another promising delivery strategy.

#### Antibody-based delivery systems

Antibodies, by virtue of their high affinity and specificity for target antigens, have been extensively harnessed as delivery vehicles. The clinical and commercial success of ADCs has validated that antibody-mediated targeting can enhance the accumulation of therapeutic payloads at disease sites while mitigating off-target toxicity [214]. This paradigm has inspired the conjugation of antibodies with PROTACs to generate degradable antibody conjugates (DACs), an approach aimed at overcoming the poor pharmacokinetics and insufficient tumor penetration that often limit the therapeutic window of small-molecule PROTACs.

**Fig. 5:**
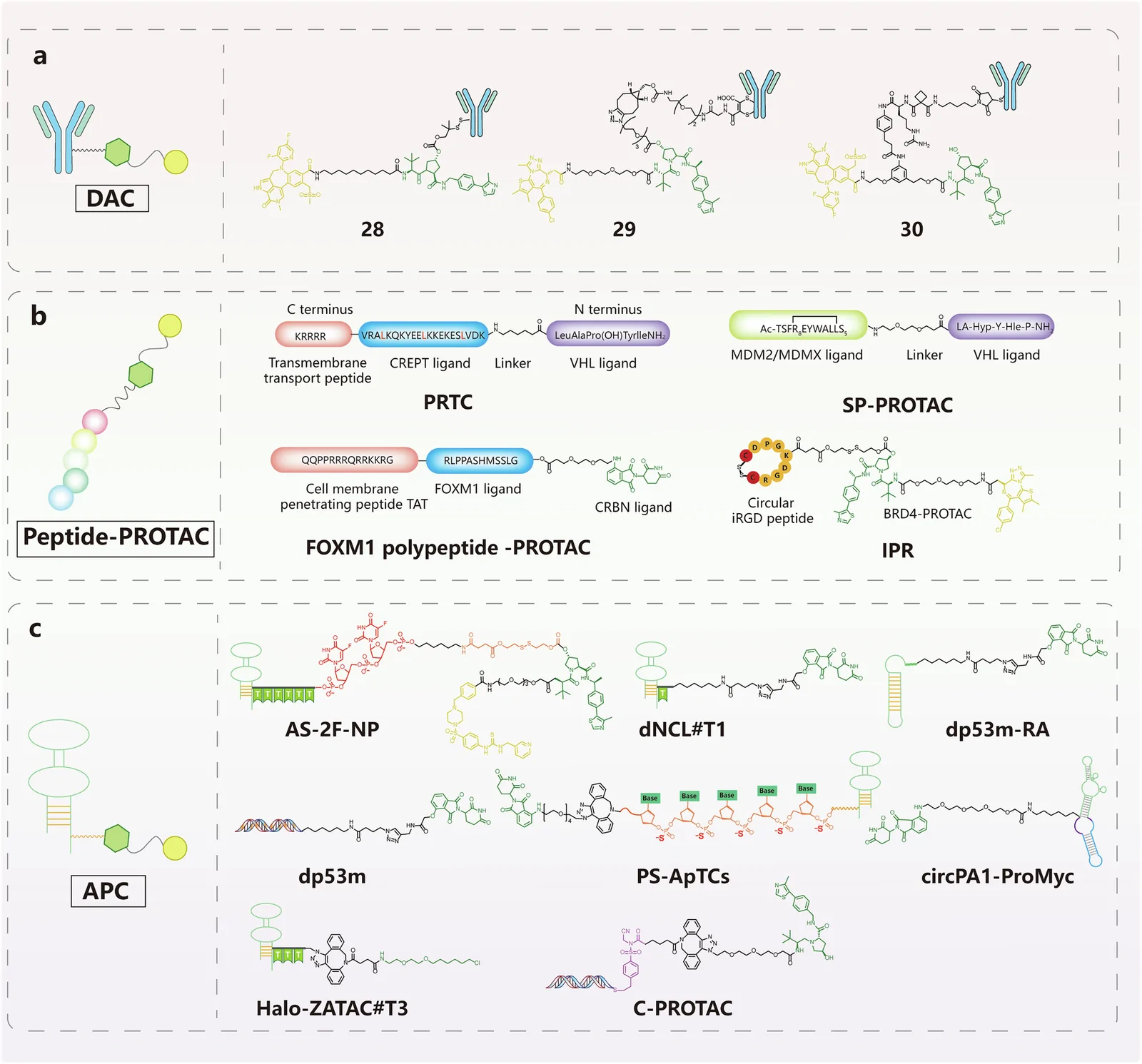
The chemical structures and compositions of the macromolecular PROTAC delivery system. These platforms solve the delivery challenges of PROTACs through different mechanisms: **a** Antibody-based degradation agents-antibody conjugates (DACs) use antibodies for cell-specific targeting; they undergo receptor-mediated endothelial cell action, and the active PROTAC payload passes through the lysosomes of the linker. Crack interpretation release. **b** The peptide-PROTAC delivery system uses the unique physicochemical properties of peptides to improve the solubility, targeting, cell absorption and bioavability of PROTAC. **c** Aptamer-PROTAC conjugates (APC) uses directional agents for targeted delivery; during cell endocylation, high concentrations of intracellular glutathione (GSH) cut the disulphide bond in the linker and release active PROTAC to induce targeted protein degradation. Overall, these systems embody the strategy of achieving targeted and efficient intracellular delivery of PROTAC molecules.

The mechanism of DAC action closely parallels that of ADCs. Following specific recognition of cell surface antigens, the conjugate undergoes receptor-mediated endocytosis and traffics to lysosomes. In the acidic lysosomal environment, the linker is cleaved to release the active PROTAC, which subsequently recruits an E3 ubiquitin ligase and the target protein (POI) to form a ternary complex, leading to POI ubiquitination and proteasomal degradation (Fig. 5a) [215]. The inaugural DAC **28** (Fig. 5a) was reported by Pillow and colleagues, who conjugated the BRD4-targeting PROTAC GNE-987 to a C-type lectin domain family 12 member A (CLL1) antibody via a methanethiosulfonate (MTS) group, demonstrating that this strategy markedly improves the pharmacokinetic profile and degradation efficiency of the PROTAC [216].

Subsequent work has expanded the DAC platform. Maneiro’s team employed copper-free click chemistry (SPAAC) to conjugate the BRD4 degrader ARV-771 to trastuzumab, yielding a HER2-targeting DAC that exhibited selective BRD4 degradation and robust stability in HER2^+^ cells [217]. Dragovich and collaborators further refined DAC **29** (Fig. 5a) design through systematic optimization, conjugating ERα-targeting PROTACs to anti-HER2 antibodies and demonstrating that modulation of linker composition and attachment sites enhances in vivo stability [218–220]. By comparing conjugates directed against STEAP1 or CLL1 with BRD4 degraders, they confirmed that DACs achieve antigen-dependent, cell type-specific protein degradation. Notably, optimized conjugates such as STEAP1-5a and CLL1-5b, designated as DAC **30** (Fig. 5a), displayed potent antitumor activity and substantially reduced systemic toxicity in murine models of prostate cancer and acute myeloid leukemia relative to free PROTAC.

Despite these advances, the development of DACs faces substantial hurdles. Over 20 DAC candidates are currently in preclinical research, with only Orum Therapeutics’ ORM-5029 and ORM-6151, along with AbbVie’s ABBV-787, having entered clinical trials. A key challenge stems from the structural complexity of conjugating a large antibody (~150 kDa) with a multifunctional PROTAC, often generating heterogeneous species that are difficult to optimize. This large size can impede diffusion within the dense tumor extracellular matrix, frequently restricting accumulation to perivascular regions and preventing penetration into hypoxic, acidic tumor cores, a limitation that contributes to heterogeneous therapeutic responses [221]. More fundamentally, an intrinsic efficiency bottleneck exists in the DAC mechanism of action. While lysosomal cleavage is designed to liberate the PROTAC, the molecule must subsequently escape from endolysosomal compartments into the cytoplasm to engage the E3 ligase machinery. Currently, this endosomal escape process remains highly inefficient, representing a critical barrier to achieving potent and sustained target degradation (Table 2) [222, 223].

#### Peptide-based delivery systems

Compared with DAC, peptide-based carriers offer advantages such as lower molecular weight and reduced immunogenicity, positioning them as promising alternatives for PROTAC delivery. They retain the functional diversity of larger proteins while circumventing major drawbacks like poor tissue penetration [224]. To address these permeability challenges, several innovative strategies have emerged. Ma et al. developed a cell-penetrating peptide-PROTAC (**PRTC**) (Fig. 5b) targeting the tumor protein CREPT by fusing a cationic pentapeptide membrane translocation sequence (KRRRRR) to the C-terminus, which enhanced transmembrane delivery and enabled effective degradation of endogenous CREPT in pancreatic cancer cells [225]. Chen et al. employed stapled peptide technology to construct **SP-PROTACs** (Fig. 5b), combining improved cellular uptake and proteolytic stability with potent TPD [226]. Meanwhile, Wang’s group focused on the transcription factor FOXM1 by conjugating a FOXM1 antagonist peptide, the E3 ligase ligand pomalidomide, and the TAT cell-penetrating peptide, yielding a **polypeptide-PROTAC** (Fig. 5b) capable of intracellular FOXM1 degradation and significant tumor inhibition [227]. To overcome barriers specific to solid tumors, He et al. integrated the tumor-penetrating and -targeting cyclic iRGD peptide with a PROTAC to form **IPR** (Fig. 5b), enhancing drug delivery efficiency in breast cancer models [228].

Despite their advantages, peptide-based delivery systems face notable limitations. Their structural simplicity, compared to antibodies, often results in weaker targeting specificity, which may lead to off-target accumulation and increased toxicity [229]. Moreover, although certain peptides exhibit cell-penetrating properties, their translocation efficiency remains generally low, particularly across stringent biological barriers such as the BBB, restricting their therapeutic applicability in hard-to-treat diseases (Table 2).

### Nucleic acid-based delivery systems

Aptamers, synthetic single-stranded DNA or RNA oligonucleotides that fold into defined three-dimensional architectures enabling high-affinity target recognition, have emerged as versatile modules for constructing targeted PROTAC delivery systems [230–232]. Their advantageous properties, including low molecular weight, minimal immunogenicity, facile chemical synthesis, robust tissue penetration, and an expansive target scope, position them as compelling alternatives to antibodies for addressing the cardinal limitations of PROTACs, namely poor selectivity, off-target toxicity, and inadequate delivery efficiency (Fig. 2d) [233].

The viability of aptamer-PROTAC conjugate (APC) has been established through successive innovations. Pioneering work by He et al. directly conjugated the nucleolin-targeting aptamer AS1411 with the BET PROTAC MZ1; the resultant APC, APR, exhibited enhanced degradation activity in breast cancer cells, albeit with the constraint of modest drug loading inherent to direct conjugation [234]. To circumvent this bottleneck, the same group ingeniously exploited the structural similarity between fluorouridine (Fdu) and thymidine, incorporating Fdu during solid-phase synthesis to enable subsequent TME-responsive carbonate linkage with a synergistic PROTAC, yielding **AS-2F-NP** (Fig. 5c) with markedly improved payload and reduced systemic toxicity [235]. This principle has been extended and refined across diverse targets. Chen’s team developed a nucleolin-targeting APC, dNCL#0, and after linker optimization to yield **dNCL#T1** (Fig. 5c), introduced a photocaged variant (opto-dNCL#T1) for spatiotemporally controlled degradation upon UV irradiation [236]. Kong et al. initially developed an RNA-based APC targeting mutant p53 (**dp53m-RA**) (Fig. 5c) but encountered limited bioavailability; they subsequently employed a molecular dynamics-driven computational strategy to guide SELEX optimization, yielding a superior DNA-based aptamer (**dp53m**) (Fig. 5c) with potent in vivo activity against p53-R175H mutant tumors [237, 238]. Addressing stability and targeted degradation of oncoproteins, Liu et al. incorporated phosphorothioate-modified backbones into CRBN ligands to create **PS-ApTCs** (Fig. 5c), enhancing nuclease resistance and enabling selective degradation of NSP1 [239]. In targeting c-Myc, Zhao’s group first identified the specific aptamer MA9C1 via high-throughput screening to construct ProMyc, and further engineered a circular chimera, **circPA1-ProMyc** (Fig. 5c), by fusing it with a cyclized PD-L1 aptamer, achieving pronounced tumor regression in xenograft models and advancing translational potential [240].

Beyond their role as delivery vehicles, aptamers have expanded the PROTAC toolkit by serving as novel E3 ligase recruiters. Yang et al. identified a non-inhibitory aptamer against the CRL2ZYG11B complex, employing it as an “E3 warhead” to construct dual-function ZATACs capable of degrading multiple targets, e.g., HaloTag (**Halo-ZATAC#T3**) (Fig. 5c), NCL, SOX2, p53-R175H, thereby unlocking access to non-canonical E3 ligases for TPD [84]. Concurrently, Huang’s team developed covalent PROTACs (**C-PROTACs**) (Fig. 5c) by linking a ZBP1-specific DNA aptamer to an E3 ligand via an N-acyl-N-alkyl sulfonamide linker; this design stabilizes the ternary complex through covalent bond formation, potently promoting ZBP1 ubiquitination and suppressing necroptosis and inflammation [241].

Despite their programmability, high affinity, and synthetic accessibility, the clinical translation of aptamer-based modalities is tempered by significant in vivo challenges. Chief among these is serum instability; unmodified oligonucleotides are rapidly degraded by plasma nucleases, resulting in half-lives of mere minutes and precluding effective target engagement [242]. Their relatively low molecular weight, while beneficial for tissue penetration, also facilitates rapid renal clearance, curtailing plasma persistence. Although PEGylation can extend circulation, it risks eliciting immunogenicity and the accelerated blood clearance phenomenon [243]. Furthermore, the mechanisms governing aptamer internalization remain poorly defined and inefficient; unlike antibody-mediated endocytosis, cellular uptake is highly variable, depending critically on the intrinsic trafficking of the target protein or on non-specific pinocytosis, representing a major bottleneck in achieving therapeutically relevant intracellular concentrations of the PROTAC payload (Table 2) [244].

## Exploration and integration of novel delivery strategies

Although prodrug-based, nanocarrier-based, protein-based and polymer-based delivery systems significantly improve the bioavailability of PROTAC and provide important strategies to overcome inherent limitations, such as high molecular weight, low cell permeability, out-target effects and unfavorable pharmacokinetic properties. However, these methods still often face problems, including limited targeting accuracy, insufficient adaptation to complex physiological microenvironments (such as hypoxia, acidity, high reduction rate), and poor ability to cross biological barriers [245]. In order to overcome these limitations, researchers have integrated multidisciplinary strategies and developed various innovative DDS to achieve efficient, accurate and controllable delivery and release of PROTAC.

### Innovative delivery strategies to overcome specific barriers

The treatment of glioblastoma (GBM) has long been hindered by the BBB and the severely hypoxic TME [246]. Addressing this dual challenge, Jiang et al. [247] ingeniously engineered a self-oxygenating PROTAC microneedle (**SOP MN**) (Fig. 4q) system. This system features a dual-layer design: the outer layer incorporates acid-sensitive, photoactivatable ARV771 PROTAC nanoparticles (PPT7 NPs) that remain inert until triggered by the acidic tumor milieu, enabling precise, light-induced BRD4 degradation. The inner layer is loaded with bovine serum albumin-manganese dioxide nanoparticles (BM NPs), which continuously catalyze endogenous H_2_O_2_ to generate O_2_, thereby alleviating hypoxia and augmenting photodynamic therapy. This design not only bypasses the BBB for targeted intracranial delivery but also enables spatiotemporally controlled protein degradation with enhanced anti-GBM efficacy. Similarly, to address suboptimal biodistribution associated with systemic PROTAC administration, Ke Cheng’s team [248] developed a localized, sustained-release minimally invasive microneedle patch. They encapsulated ERα degrader ERD308 and CDK4/6 inhibitor palbociclib within pH-sensitive MPEG-poly(β-amino ester) micelles, which were then incorporated into biodegradable **microneedles patches** (Fig. 4r) for local transdermal delivery. This system achieves deep tumoral penetration, sustained drug release, and high local retention with favorable safety, offering a translatable strategy for localized PROTAC therapy in solid tumors like breast cancer. In a related approach targeting oligonucleotide-based PROTAC delivery across the BBB, Ge et al. [249] developed an integrated platform termed **FRONTAC** (Fig. 4s). Leveraging rolling circle amplification, they synthesized DNA nanoflowers with high cargo capacity and functionalized them with transferrin receptor aptamers for brain-targeted delivery. The platform enabled efficient loading of an oligo-PROTAC (DFUS-PS9C), conjugated via click chemistry to an E3 ligase ligand, facilitating targeted ubiquitination and degradation of FUS protein, a strategy with significant implications for PROTAC-based therapies in neurodegenerative diseases.

### Construction of modular self-assembling and smart-responsive nanoplatforms

The conventional design of PROTACs is often encumbered by synthetic complexity and fixed ligand stoichiometry, which can lead to suboptimal degradation efficiency. To address this, Yang et al. [250] pioneered a “Split and Mix” PROTAC (**SM-PROTAC**) (Fig. 4t) nanoplatform wherein monomeric units bearing either the POI or E3 ligase ligand self-assemble into nanostructures directly within cells. This dynamic self-optimization of ligand stoichiometry enables precise tuning for maximal degradation efficacy. Despite its high programmability, the platform’s therapeutic utility was initially limited by modest in vivo performance. Building on this concept, the same group integrated it with lipid technology to develop **LipoSM-PROTAC** (Fig. 4u) [251]. By encapsulating the self-assembling components within folate-functionalized liposomes, this system synergized the stoichiometric tunability of SM-PROTACs with the enhanced bioavailability, tumor targeting, and biocompatibility of lipid nanocarriers, achieving significantly improved degradation of ERα and demonstrating generalizability to other targets [252].

In parallel, alternative strategies have emerged to control PROTAC assembly through endogenous stimuli. Chen’s team [253] devised a **supra-PROTAC** (Fig. 4v) approach triggered by tumor-associated enzymes, where a peptide precursor undergoes specific cleavage and subsequent non-covalent co-assembly with ligand-conjugated peptides to form functional nano-PROTACs directly in diseased cells. This method enhances targeted delivery and opens avenues for the in situ construction of functional degraders. Addressing the “hook effect” prevalent in conventional PROTACs, Zhang et al. [254] developed a **rigid β-layer nano-PROTAC** (Fig. 4y) system assembled via a GSH-responsive click reaction in tumor cells. The resulting ordered multivalent network enables simultaneous engagement of multiple POI and E3 ligase copies, promoting sustained, dose-dependent degradation and improved tumor retention. Expanding the scope to multi-target modulation, Lu and colleagues [255] engineered **dual-target degrading NP-PROTACs** (Fig. 4w) by conjugating β-catenin and STAT3 targeting peptides to a DSPE-PEG scaffold via disulfide bonds, which self-assemble into nanoparticles. This strategy achieved potent dual-protein degradation in a colorectal cancer model, yielding synergistic antitumor effects. Notably, it also remodeled the tumor immune microenvironment by enhancing CD103^+^ dendritic cell infiltration and cytotoxic T cell activation, thereby counteracting immunosuppression and underscoring the promise of nano-PROTACs in cancer immunotherapy.

### Molecular engineering and the exploration of novel mechanisms

Enhancing the cellular permeability of PROTACs and circumventing efflux pump-mediated resistance are critical determinants of their in vivo efficacy. To address this, Ciulli and co-workers [256] introduced ferrocene as a structurally dynamic linker, capitalizing on its inherent “chameleonicity” to develop a class of degraders termed **FerroTACs** (Fig. 4x), e.g., AS7. The ferrocene moiety, characterized by a freely rotating structure around its central iron ion, enables the molecule to dynamically adapt its conformation in response to the surrounding solvent environment [257, 258]. This conformational plasticity confers superior membrane permeability and significantly reduces the compounds’ recognition by efflux pumps such as P-glycoprotein (P-gp). Consequently, FerroTACs exhibit enhanced cellular accumulation and potent degradation activity, outperforming conventional PROTACs in certain models and offering a strategic insight into designing degraders with improved pharmacokinetic profiles. Shifting the focus from general permeability to subcellular localization, Mo et al. [259] tackled the challenge of targeting membrane proteins by developing a membrane-anchored intracellular E3 ligase strategy (**MembTAC**) (Fig. 4z). This modular system integrates a pH-sensitive peptide (pHLIP) that facilitates TME-specific membrane insertion under acidic conditions. The intracellular C-terminus of the anchored peptide recruits the E3 ligase CRBN, while a replaceable N-terminal recognition module binds the extracellular domain of the target membrane protein. This design effectively tethers the intracellular degradation machinery to the inner leaflet, enabling ubiquitination and degradation of the target’s intracellular domain and thereby extending PROTAC applicability to traditionally “undruggable” membrane proteins. In a distinct approach targeting hepatocellular carcinoma (HCC) cancer stem cells (CSCs), Wang’s team [260] engineered a **STAT3 degradation prodrug system** (Fig. 4aa) for liposomal delivery. The construct, inS3-TEG-VL-TK, links a STAT3-binding domain (inS3) to a caged E3 ligase ligand (VL-TK) via an oligoethylene glycol (OEG) linker and a thioketal (TK) moiety. Encapsulated within cRGD-modified cationic liposomes to ensure CSC targeting and lysosomal escape, the prodrug is activated specifically in the reactive oxygen species (ROS)-rich CSC environment upon thioketal cleavage. The liberated active PROTAC then degrades STAT3, leading to the attenuation of CSC stemness and, notably, the reprogramming of the immunosuppressive TME toward an immune-supportive state. This work underscores the therapeutic versatility of PROTAC prodrugs, demonstrating their potential not only for TPD but also for immunomodulatory intervention in cancer.

### Synergistic and mechanism-driven delivery chemistry strategies

The integration of platinum(IV) prodrug technology with PROTACs, exemplified by Li et al.’s design of the conjugate CW-2, represents a strategic advance in overcoming the limitations of conventional platinum-based chemotherapy, such as toxicity and resistance [261–265]. Leveraging the octahedral hexacoordinate structure of Pt(IV), these prodrugs exhibit enhanced metabolic stability and offer modifiable axial ligands for fine-tuning pharmacological properties [266–268]. **CW-2** (Fig. 4ab) concurrently induces DNA damage via the Pt(IV) moiety and suppresses DNA repair pathways through the PROTAC component, resulting in potent synergistic anticancer effects and improved membrane permeability, offering a viable strategy to circumvent platinum resistance. Concurrently, mechanistic insights from Wang’s team have elucidated that the cellular uptake of large or highly polar beyond-Rule-of-5 (bRo5) molecules is mediated by CD36-dependent endothelial cell uptake, leading to the proposal of a “chemical endothelial cell drug chemistry” (CEMC) strategy [269]. This approach rationally designs PROTACs to enhance CD36 receptor binding, thereby facilitating transcellular transport and holding promise for improving the bioavailability and in vivo efficacy of bRo5 therapeutics.

Current innovations in PROTAC delivery systems are steering the field toward modular, intelligent, and collaborative platforms. Modular self-assembly strategies, such as SM-PROTAC, LipoSM-PROTAC and Supra-PROTAC, offer programmable and dynamically adjustable architectures, particularly advantageous when optimal ligand stoichiometry is unpredictable or when temporal optimization of PROTAC function is required. Nevertheless, challenges remain, including potential interference from the intracellular environment on precursor assembly, heterogeneity in nanostructure formation, and rapid renal clearance of low-molecular-weight precursors. Furthermore, the in vivo fate of assembled nanostructures and potential immunogenicity from peptide or nucleic acid-based modules warrant careful consideration [270–274]. Joint delivery strategies aim to achieve combinatorial synergy by co-delivering PROTACs with complementary therapeutic agents or by enabling multi-target degradation. However, their success hinges on precise spatiotemporal coordination of component release. Intelligent, stimuli-responsive delivery systems have improved targeting accuracy and reduced off-target toxicity, yet the structural and functional integrity of the released PROTAC must be rigorously confirmed to ensure efficient ternary complex formation [77, 172, 275]. Future efforts should therefore focus on improving assembly fidelity, release coordination, long-term stability, and biosafety, so as to facilitate the translation of these advanced delivery concepts toward practical therapeutic applications.

## Summary and outlook

PROTACs have emerged as a transformative modality in TPD, yet their clinical translation is increasingly contingent upon innovations in delivery systems. A range of carriers, including LNPs, polymer-based nanoparticles, and ADCs, has been shown to markedly enhance the cellular uptake, pharmacokinetic profiles, and degradation efficacy of PROTAC molecules. By employing physical encapsulation or chemical conjugation, these platforms circumvent the physicochemical limitations inherent to many PROTACs, thereby liberating medicinal chemistry efforts from the empirical correction of poor drug-like properties and enabling a sharper focus on optimizing the ternary complex for maximal degradation activity [56, 276–278]. Taken together, delivery systems are not merely auxiliary tools for formulation optimization, but have become an essential design dimension for realizing the therapeutic potential of PROTACs. Among current approaches, chemically engineered prodrug strategies, smart nanocarrier-based systems, and targeted conjugate platforms appear particularly promising for improving bioavailability, tissue selectivity, and controlled intracellular release. Despite these advances, however, current delivery strategies are not without limitations. The immunogenic potential of nanocarriers can attenuate therapeutic efficacy [113], while protein- or peptide-based vectors are susceptible to enzymatic degradation and premature drug release, leading to diminished on-target effects and heightened off-target risks. Nucleic-acid-based carriers, in turn, suffer from instability and rapid systemic clearance, and may provoke unintended immune responses [279]. Moreover, the preponderance of studies remains confined to in vitro models, underscoring a pressing need for systematic in vivo investigations to validate clinical translatability under physiologically relevant conditions.

Looking forward, the evolution of PROTAC delivery is being shaped by multidimensional innovations, among which the integration of artificial intelligence (AI) and computational modeling holds particular promise. Successful PROTAC design demands the concurrent optimization of multiple attributes, ternary complex stability, cellular permeability, pharmacokinetics, and tissue selectivity, a challenge for which AI is uniquely suited given its capacity to process high-dimensional, heterogeneous datasets [280, 281]. Traditional discovery pipelines, reliant on iterative ligand modifications and empirical screening, are resource-intensive and serendipitous. By contrast, deep-learning-based generative models can now interrogate large chemical-biological datasets to uncover structure-activity relationships, generating novel scaffolds with plausible three-dimensional conformations and tailored engagement of both target and E3 ligase, thereby vastly expanding accessible chemical space [62, 282–287]. For instance, DeepTernary, developed by the Deng lab at UCLA, enables accurate prediction of PROTAC-induced ternary complex structures to guide linker design [288]; complementary tools such as AIMLinker, Chemistry42, and LM-PROTAC facilitate the rapid generation of molecules satisfying multiple design constraints [289–291]. AI also extends its utility to delivery strategy design: in antibody-PROTAC conjugates, for example, machine learning models trained on ADC data can predict linker stability and payload release kinetics, enabling rational design of controlled-release switches. Platforms like ProteinQure have leveraged this approach to advance PQ2033, a peptide-drug conjugate now in clinical development [292]. Collectively, these efforts point toward an end-to-end rational design ecosystem that seamlessly integrates target validation, molecular generation, property prediction, and candidate prioritization [293].

Beyond AI-driven design, the delivery platforms themselves are diversifying along several innovative trajectories. Stimuli-responsive carriers, engineered to exploit pH gradients, enzymatic activity, or light, offer spatiotemporally controlled release, mitigating the “hook effect” associated with premature drug liberation and enabling precise modulation of dose-response relationships [294]. Multifunctional combination platforms, capitalizing on high loading capacities, permit co-delivery of PROTACs with chemotherapeutics, epigenetic modifiers, or immunomodulators, thereby overcoming resistance through synergistic multitarget engagement [295]. Moreover, advances in barrier penetration are poised to unlock applications in CNS disorders: bioinspired vectors harnessing endogenous transport mechanisms now demonstrate promise in crossing the BBB, opening new avenues for targeted degradation in neurodegenerative diseases [112, 296–303]. Overall, stimulus-responsive systems, targeted conjugate strategies, multifunctional combination platforms, and BBB-penetrating delivery vehicles represent some of the most promising directions for future translational development. Despite this conceptual diversity, the overarching objective remains convergent, enhancing bioavailability, minimizing off-tissue toxicity, and sharpening degradation specificity to expedite clinical translation. Nevertheless, critical hurdles persist: the rapid evolution of PROTAC architectures demands delivery platforms with modular reconfigurability; scale-up economics of carrier materials may impede industrial adoption; and excipient-related immunogenicity necessitates rigorous biocompatibility assessments. In addition, insufficient in vivo validation, incomplete understanding of intracellular trafficking and endosomal escape, and the difficulty of balancing release efficiency with tissue selectivity remain major unresolved challenges in the field.

Importantly, advances in PROTAC delivery are poised to resonate broadly across the TPD landscape. Emerging modalities such as lysosomal-targeting chimeras, autophagy-targeting chimeras, and phosphorylation-inducing chimeras share analogous bifunctional designs and confront similar in vivo barriers [304–307]. Consequently, insights gleaned from PROTAC delivery optimization are directly transferable, accelerating the development of a versatile toolkit for precision degradation therapies.
